# Harnessing Innovative Technologies to Train Nurses in Suicide Safety Planning With Hospital Patients: Formative Acceptability Evaluation of an eLearning Continuing Education Training

**DOI:** 10.2196/56402

**Published:** 2024-09-06

**Authors:** Doyanne Darnell, Andria Pierson, Michael J Tanana, Shannon Dorsey, Edwin D Boudreaux, Patricia A Areán, Katherine Anne Comtois

**Affiliations:** 1 Department of Psychiatry & Behavioral Sciences University of Washington Seattle, WA United States; 2 Lyss.io Seattle, WA United States; 3 Department of Psychology University of Washington Seattle, WA United States; 4 Department of Emergency Medicine University of Massachusetts Chan Medical School Worcester, MA United States

**Keywords:** suicide prevention, hospital, training, e-learning, artificial intelligence, AI, task-shifting, quality assessment, fidelity, acceptability, feasibility, eLearning, suicide, quality, innovative, nurse, education training, safety planning, pilot study, virtual patient, web-based, role-play, microcounseling skills, United States

## Abstract

**Background:**

Suicide is the 12th leading cause of death in the United States. Health care provider training is a top research priority identified by the National Action Alliance for Suicide Prevention; however, evidence-based approaches that target skill building are resource intensive and difficult to implement. Novel computer technologies harnessing artificial intelligence are now available, which hold promise for increasing the feasibility of providing trainees opportunities across a range of continuing education contexts to engage in skills practice with constructive feedback on performance.

**Objective:**

This pilot study aims to evaluate the feasibility and acceptability of an eLearning training in suicide safety planning among nurses serving patients admitted to a US level 1 trauma center for acute or intensive care. The training included a didactic portion with demonstration, practice of microcounseling skills with a web-based virtual patient (*Client Bot Emily*), role-play with a patient actor, and automated coding and feedback on general counseling skills based on the role-play via a web-based platform (*Lyssn Advisor*). Secondarily, we examined learning outcomes of knowledge, confidence, and skills in suicide safety planning descriptively.

**Methods:**

Acute and intensive care nurses were recruited between November 1, 2021, and May 31, 2022, to participate in a formative evaluation using pretraining, posttraining, and 6-month follow-up surveys, as well as observation of the nurses’ performance in delivering suicide safety planning via standardized patient role-plays over 6 months and rated using the Safety Plan Intervention Rating Scale. Nurses completed the System Usability Scale after interacting with Client Bot Emily and reviewing general counseling scores based on their role-play via Lyssn Advisor.

**Results:**

A total of 18 nurses participated in the study; the majority identified as female (n=17, 94%) and White (n=13, 72%). Of the 17 nurses who started the training, 82% (n=14) completed it. On average, the System Usability Scale score for Client Bot Emily was 70.3 (SD 19.7) and for Lyssn Advisor was 65.4 (SD 16.3). On average, nurses endorsed a good bit of knowledge (mean 3.1, SD 0.5) and confidence (mean 2.9, SD 0.5) after the training. After completing the training, none of the nurses scored above the expert-derived cutoff for proficiency on the Safety Plan Intervention Rating Scale (≥14); however, on average, nurses were above the cutoffs for general counseling skills per Lyssn Advisor (empathy: mean 4.1, SD 0.6; collaboration: mean 3.6, SD 0.7).

**Conclusions:**

Findings suggest the completion of the training activities and use of novel technologies within this context are feasible. Technologic modifications may enhance the training acceptability and utility, such as increasing the virtual patient conversational abilities and adding automated coding capability for specific suicide safety planning skills.

**International Registered Report Identifier (IRRID):**

RR2-10.2196/33695

## Introduction

### The Need to Address Suicide Risk Among Hospitalized Patients

Suicide is the 12th leading cause of death in the United States [1] with nearly 49,500 estimated deaths in 2022 [2]. Among those who die by suicide in the United States, the majority have contact with the health care system, including general medical and acute care settings, in the year before their death [3,4]. Medically hospitalized patients, such as those admitted for chronic and severe medical conditions or traumatic injury, are known to have comorbid or preinjury suicide risk factors, such as behavioral health conditions [5,6]. Further, traumatic events and stressful experiences during hospitalization (eg, being treated in an intensive care unit) are known to increase risk factors for suicide, such as posttraumatic stress disorder and depression [7,8], and studies show an increased risk of suicide after traumatic injury hospitalization [9,10]. Integrating suicide preventive interventions into acute medical care settings for patients identified as at risk of suicide holds promise for reducing the rate of suicide and is consistent with goals set in the National Strategy for Suicide Prevention [11].

The Joint Commission, which provides oversight, standards, and guidelines for health care organizations nationally, recommends suicide risk screening followed by interventions to address safety concerns for patients seen in medical settings among populations at greater risk of suicide [12]. One recommended intervention is suicide safety planning, a brief, evidence-based preventive intervention that may be feasible to deliver with at-risk acute care patients to support patients after hospitalization [13-16]. Suicide safety planning includes a provider working collaboratively with a patient to identify a multistep plan for coping with suicidal thinking and urges to get safely through or avert a suicidal crisis. Once adequately trained, suicide safety planning can be completed by a diverse array of health care workers, such as nurses, physicians, and medical social workers, and does not require behavioral health expertise to deliver.

### The Role of Acute and Intensive Care Nurses in Suicide Prevention

Acute and intensive care bedside nurses may be ideally positioned to engage patients in suicide prevention activities, such as suicide safety planning. Bedside nurses commonly conduct the suicide risk screening for medically hospitalized patients in those hospitals implementing screening protocols and are, therefore, already engage in conversations with patients about suicide and help to ensure their safety during hospitalization [17]. Bedside nurses also spend considerable time with patients and have the opportunity to build trusting collaborative relationships with patients over the course of a hospital stay, which may be conducive to collaborative safety planning [18]. Nurses also routinely help patients prepare for discharge and teach patients about how to care for their postdischarge medical needs; suicide safety conversations may fit into these postdischarge topics.

Historically, health care workers have not received training in suicide prevention through their professional programs, and research with nurses working with nonpsychiatric populations indicates many nurses experience discomfort asking patients about suicide, talking about suicidality with patients, and knowing how to effectively respond when they do learn of a patient’s suicidality [19-21]. Recognizing the need for training, some states now require a variety of medical professionals to complete continuing education on suicide and suicide prevention [22]. There has been a proliferation of evidence-based continuing education trainings (eg, the Question, Persuade, and Refer Gatekeeper training and LivingWorks Applied Suicide Intervention Skills training for safety planning [23,24]). All trainings provide some didactics regarding facts about suicide; however, some trainings, such as *gatekeeper* programs, are geared toward persons across a variety of settings (eg, schools and workplaces) who may be in a position to identify warning signs of suicide, screen for suicidality, and refer people to appropriate care. These training courses are geared toward clinicians, health care workers, or persons in roles in which they would be able to help mitigate risk of suicide and offer evidence-based interventions to reduce suicidality or suicidal behavior. It is rare for any of the continuing education trainings to include opportunities for trainees practice skills and receive feedback on this practice [25,26], despite the evidence-base for such methods [27,28]. Further, although continuing education programs in suicide prevention are associated with improved self-reported competence, knowledge, and suicide-related attitudes, there are limited data on how these trainings impact skill building or actual skills used in practice [26,29-32].

### Using Technology to Enhance the Scalability and Sustainability of Continuing Education in Suicide Prevention

The primary barrier to offering trainees opportunities for skills practice with feedback is that traditional methods for doing so are labor intensive and not scalable, requiring considerable time from expert trainers [28,33,34]. Even if skills practice is feasible in continuing education contexts which aim to reach large audiences (eg, having trainees pair up), it is not practicable for the instructor to observe trainee skills, systematically assess the quality of skills based on this observation, and then provide tailored, expert feedback to guide skill building. Novel computer technologies, often harnessing artificial intelligence, are now available, which hold promise for increasing the feasibility of providing trainees opportunities across a range of continuing education contexts to engage in skills practice with constructive feedback on performance [35,36]. Although such technologies can be costly to develop initially, they can be highly pragmatic and cost-effective for subsequent deployment [37].

For instance, platforms exist in which trainees can interact and practice skills for working with patients across a variety of health care situations with a *virtual patient*. Virtual patients are defined as “interactive computer simulations of real-life clinical scenarios for the purpose of health professions training, education, or assessment” and are known to improve knowledge and skills across a variety of health profession education [38]. Such platforms can use either preprogrammed content (ie, multiple-choice options for the trainee and preprogrammed responses for the virtual patient based on the multiple-choice selection [39]) or allow both the trainees and virtual patients to generate natural language questions and responses using artificial intelligence [40]. Benefits to virtual patients is that they can be accessed anytime from any location with access to a computer, can offer greater confidentiality for the trainee, require time only from trainee, are highly standardized, and can be programmed to target training for various patient presentations (eg, range of severity of suicidal thoughts).

With regard to suicide prevention, a virtual patient platform using preprogrammed content demonstrated improvement in self-efficacy and preparedness among college students trained to be gatekeepers (people trained to identify others at potential risk of suicide and refer for additional assessment or care) in a randomized clinical trial [41]. In addition, the training impacted gatekeeper behavior, resulting in higher rates of students being referred for mental health services by the college students trained to be gatekeepers. There is emerging evidence that virtual patients are as effective as engaging in role-play with human actors or peers pretending to be a patient experiencing suicidality. A recent quasi-experimental study with graduate social-work students compared the impact of a naturalistic peer-to-peer role-play versus training with a virtual patient on a variety of clinical skills for working with military personnel, including student responses to the virtual patient’s report of experiencing suicidality (whether the virtual patient used a preprogrammed approach or allowed for spontaneous language generation was not specified) [42]. Findings indicated similar performance regardless of whether the students were trained using role-play or the virtual patient across clinical skills based on a 20-minute standardized patient role-play examination. Other promising examples of virtual patients include pilot research with a virtual client to train students with a Master of Social Work degree, specializing in suicide prevention—including suicide safety planning—that uses multiple-choice role-play with video-recorded human actors prerecorded to respond appropriately to student’s choices that demonstrated feasibility and acceptability [43]. The same technology demonstrated feasibility and acceptability in training practitioners from a Federally Qualified Health Center in suicide risk assessment [44]. In response to the growing demand for crisis counselors, the Trevor Project, an organization with the mission to end suicide among young lesbian, gay, bisexual, transgender, queer, and others people, has partnered with Google.org to create an artificial intelligence simulation called the Crisis Contact Simulator that allows crisis counselors to practice interacting with callers to help them through a suicidal crisis [45]. The Crisis Contact Simulator can be modified to create a variety of personas so that counselors can practice with people representing diverse backgrounds, situations, and nature of crises.

Artificial intelligence technology can be used to evaluate trainee performance within the context of a human-to-human role-play. For instance, this study uses a platform (Lyssn.io [46]) that was designed to capture video or audio recordings of psychotherapy or counseling sessions, convert these to transcript, and then automatically computer-generate scores for counseling skills, which are then provided as feedback to the counselor of their performance. Extensive developmental research with this platform demonstrates its ability to accurately code general counseling skills, such as empathy, collaboration, and the use of open-ended questions and reflections and found to be usable by psychotherapists [47-49]. The capacity for automated coding of human-to-human interaction is critical if we are to understand how trainees perform in not only practice but in actual interactions with patients.

### This Study

This study is part of Project WISE (Workplace Integrated Support and Education), which includes pilot and developmental research to explore the role of and opportunities for nurses to engage in suicide prevention activities with medically hospitalized patients [50]. Given that acute and intensive care nurses may be in a position to not only screen and refer patients for hospital-based, suicide-related services but could also engage patients in a brief, evidence-based preventive intervention at some point over the course of their hospital stay, we conducted a formative evaluation of an eLearning continuing education training for nurses in suicide safety planning. The training incorporates virtual patient and automated coding technology to facilitate opportunities to engage in counseling skills practice with feedback on their skills. The primary objectives of this pilot study were to evaluate the feasibility and acceptability of the eLearning training and engagement with the components and technologies. Secondarily, we examined learning outcomes, such as nurses self-report of knowledge and confidence in suicide safety planning skills and the quality of observed skills used in role-plays with a patient actor over time as well as nurses’ intentions to use skills learned with their patients.

## Methods

### Ethical Considerations

The study was approved by the University of Washington Institutional Review Board (STUDY00013577). All participants completed informed consent procedures. Steps were taken to protect participant privacy and confidentiality, including secure storage of data, use of US Health Insurance Portability and Accountability Act–compliant videoconferencing software and video data storage, and deidentification of survey and coded qualitative data for analysis and retention. Persons from vulnerable populations were not recruited. Participants received compensated for research activities totaling up to US $400.

### Design and Procedure

**Figure 1:**
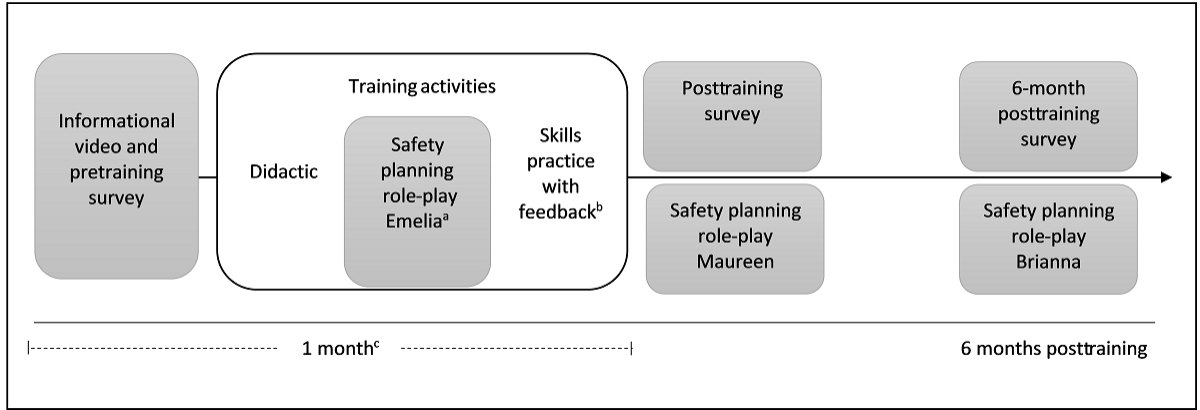
Participant flow through the formative evaluation research and training activities over time. Shaded boxes indicate activities used to evaluate the training. The formative evaluation was completed with 18 nurses working with medically hospitalized patients. Training activities were planned to be completed over the course of 1 month and research activities over the course of 6 months. ^a^The Emelia role-play was used to observe the quality of nurses’ suicide-safety planning skills after the didactic and demonstration portion of the training; however, the role-play also provided nurses an opportunity to practice suicide safety planning, which may have training benefits. ^b^Nurses first practiced with Client Bot Emily and then completed the Natalie role-play with the standardized patient actor; automated feedback from Lyssn Advisor was provided for their performance on the Natalie role-play. ^c^The training activities were expected to be feasibly completed within 1 month.

We conducted the formative evaluation using pre- and posttraining surveys, observational data on training engagement, and observation of nurse performance delivering suicide safety planning via standardized patient role-plays over time (Figure 1).

The first author worked with acute and intensive care inpatient unit nurse managers to facilitate recruitment, which included a combination of attending a nurse daily meeting to advertise the study and hand out recruitment flyers, asking the nurse manager to email a study recruitment flyer to unit nurses and post a flyer in work areas. Recruitment occurred between November 1, 2021, and May 31, 2022. Nurses contacted the study team to express interest in the study. Participants completed a web-based informed consent process by reviewing the consent form via a survey and typing their name into a box at the bottom of the consent form and submitting the form to indicate consent. They were provided with an informational video to watch that described the study activities and the study team contact information for questions. All training and research activities were voluntary and completed outside of work hours, with an anticipated 3 to 4 hours for training and 3 additional hours for research activities over the course of 6 months. Nurses were remunerated for all activities: US $25 for watching the orientation video, US $25 per survey, US $50 per role-play, and US $100 for completing the didactic training activity (described below).

### Setting

The study took place at a US level 1 trauma center, which is also a safety-net hospital and academic medical center. Nurses there are required to complete 6 hours of suicide-prevention training at least once for state licensure and universally screen all patients admitted to the emergency department and inpatient units for suicidality using the Columbia Suicide Severity Rating Scale triage version [51]. The triage screener results in designation of patients as being at *No Identified Risk*, *Low Risk*, *Moderate Risk,* or *High Risk*. Usual care for patients screening at high risk includes suicide precautions, such as ensuring that the environment is safe from lethal means; having a patient monitor sit with the patient; and notifying the medical team, who would request a consult from the hospital psychiatry service. Low- or moderate-risk patients are provided suicide-prevention resources at discharge (eg, crisis line) and may request to see a hospital social worker. At the time of the study, it was not standard practice for nurses to engage in suicide safety planning with patients.

### Description of the eLearning Training in Suicide Safety Planning

#### Design of the eLearning Training

eLearning is the application of information and communication technologies in the delivery of education services (eg, electronic instructional content, web-based instructional tools) [52]. We designed an eLearning training based on adult learning theories and findings from implementation science on effective practices for training in evidence-based interventions, with the goal of creating a brief, scalable training that could be integrated into the routine workflow of nurses (ie, able to be completed in parts using an internet-capable computer or smartphone). Both adult learning theories and findings from implementation science point to the need for trainings to include not only didactic material to build a knowledge base and understanding of how to do an intervention but also the need for trainees to practice skills and receive feedback on this practice [28,53]. Therefore, as described below, we designed a web-based training that could be completed in multiple sessions, at times that were convenient for nurses, and offered both didactic content (including demonstration of an expert delivering parts of the intervention) as well as opportunities for practice and feedback on specific and general counseling skills needed for effective delivery of suicide safety planning.

#### Web-Based Training Platform

We used the REDCap (Research Electronic Data Capture; Vanderbilt University) to create a platform to guide nurses through the training activities and complete research surveys [54]. The REDCap system sent email notifications to the nurses when it was time to complete each part of the training, which included detailed instructions and any needed materials or hyperlinks to relevant websites they would need to access to complete training activities.

#### Didactic and Demonstration Content

The didactic and demonstration portion was estimated to take 1 hour and could be completed over multiple sessions. The research team e-mailed nurses a unique link to a REDCap survey with the instructions and links needed to complete this portion of the training. The didactic portion included a 12.5-minute segment of a lecture given by Barbara Stanley, PhD, codeveloper of the Safety Planning Intervention, for the Stockholm Psychiatry Lectures series in July 2019 that described the Safety Planning Intervention and elements of the safety plan. The video was publicly available on the internet by the Karolinska Insitutet in Sweden [55]. The demonstration portion also included 4 videos of Greg Brown, PhD, codeveloper, engaging a patient in the Safety Planning Intervention, publicly available at the United States Joint Commission website [16]. The Joint Commission videos total 16.4 minutes and demonstrate obtaining a narrative of a suicidal crisis and identifying warning signs for a future crisis, identifying means of distraction that can be done on one’s own and those that involve contacting or being around other people, identifying friends and family to reach out to for help, and lethal means counseling. We asked nurses to respond to a knowledge question about each video to encourage engagement with the material.

We also provided nurses with written materials about the Safety Planning Intervention; these included (1) commentary that safety planning be done in an explicitly collaborative fashion, with an empathic and nonjudgmental style, and to use microskills of asking open-ended questions to elicit patient perspectives and making reflective statements to demonstrate understanding of patient statements; (2) a copy of a 2-page guide on safety planning created by the Safety Planning Intervention developers and published by the Western Interstate Commission for Higher Education along with a sample copy of a safety-plan template [56]; (3) a link to the Veteran’s Affairs *Safety Planning Intervention Manual* [57]; and (4) tips developed by the first author (DD) for completing the safety planning process and the safety plan that align with aspects of the Safety Planning Intervention. We asked nurses to complete 4 knowledge questions about the 2-page safety-planning guide to encourage engagement with the material. Nurses had access to these training materials via the study website throughout the course of the study and were encouraged to review these documents before completing the standardized patient role-plays.

#### Postdidactic Standardized Patient Role-Play: Emelia Persona

After completing the didactic and demonstration portion of the training, nurses completed a role-play with a patient actor whose persona was named Emelia (Multimedia Appendix 1). This role-play served the dual purpose of (1) observing for evaluation purposes nurses’ knowledge and skills in safety planning before their use of the novel technologies for skills practice and feedback and (2) providing an opportunity for nurses to practice safety planning and the role-play process. Nurses did not receive feedback on their skills based on this role-play.

#### Practice and Feedback on Counseling Microskills: Client Bot Emily

After completing the first role-play, the research team e-mailed nurses a link to a REDCap survey with instructions for practicing counseling microskills of asking open-ended questions and making reflections (eg, reflective statements of what a patient says or means and open questions to elicit a patient’s perspective and interests) using Client Bot Emily developed by Lyssn.io. Client Bot Emily is a virtual patient that uses machine learning and artificial intelligence to simulate interactions in text chat format, providing the opportunity for trainees to practice general counseling microskills and receive real-time feedback on performance and coaching on the use of these skills [58]. The feedback included notifying the trainee when they correctly or incorrectly asked open-ended questions or made reflections. Client Bot Emily’s persona as a patient experiencing suicidality was developed in partnership with Lyssn.io and the research team. This virtual patient was both trained on conversations around suicide from Reddit [59] and primed with persona information that encourage the virtual patient to steer conversations toward suicidality.

The REDCap survey included a link to the nurse’s Lyssn.io account, instructions for interacting and practicing skills with Client Bot Emily, and the System Usability Survey [60] items to complete when finished. Nurses could also write in comments at the end of the survey about their experience with Emily. Nurses were asked to complete at least 1 round (15 minutes) of interaction with Emily, which included practicing open-ended questions followed by practicing reflections. They could use background information provided about Emily’s persona in addition to her computer-generated responses to come up with their questions and reflections. Nurses could complete this training activity across multiple sessions and could return to practice with Client Bot Emily as desired throughout the course of the study. Nurses were asked to complete the activity within 1 week.

Emily’s persona is described as a 25-year-old woman hospitalized after being hit by a car when she was walking home from work through a bus intersection. She screened positive on the Columbia suicide screening when she came into the emergency department, acknowledging that she has a history of attempted suicide as a teenager. She told the nurse yesterday that she had been feeling hopeless and overwhelmed and had been having thoughts of wanting to end it all. She has been working as a server at a restaurant and will not be able to go back to serving any time soon as she recovers. She lives alone in an apartment and is distraught about what to do and does not have family nearby to help. Her boyfriend came to visit her in the hospital, but she explained this is a new relationship and she does not want to burden him with her care.

#### Standardized Patient Role-Play for Receiving Feedback: Natalie Persona

After completing the microskills practice with Client Bot Emily, nurses completed a role-play to practice their skills in suicide safety planning with a patient actor (persona name Natalie) and receive feedback on their general counseling skills of empathy and collaboration through Lyssn Advisor (described below) for this role-play.

#### Feedback on General Counseling Skills: Lyssn Advisor

The patient actor uploaded the Natalie persona role-play to the Lyssn.io website for the nurse to privately review. Lyssn Advisor, developed by Lyssn.io, uses speech signal, natural language processing, and machine learning to first convert the role-play audio content to a transcript and then assess the nurse’s quality of general counseling skills based on the transcript text. Lyssn Advisor also creates a transcript of the audio, which the nurse may review alongside their video.

The research team e-mailed nurses a link to a REDCap survey with instructions for completing the Lyssn Advisor feedback activity and the System Usability Scale (SUS) to complete afterward. The instructions for the activity included a description of the interpretation of empathy and collaboration scores based on that provided in the Motivational Interviewing Treatment Integrity Scale 3.1.1 manual [61]. Reviewing their scores through Lyssn Advisor could take as little as 5 minutes; however, nurses were told they could review their video and transcript if they choose, which could take an additional 30 minutes. Nurses could complete this activity over multiple sessions and return to it at any point over the course of the study. Nurses were asked to complete the activity within 1 week.

### Demographics Questionnaire

Nurses completed demographics questions as part of the pretraining survey, including, age, gender (transgender, female, male, nonbinary, or other), race and ethnicity (Hispanic or Latino, African American or Black, American Indian or Alaska Native, Asian, Caucasian or White, Native Hawaiian or Pacific Islander, or other), whether they work in the intensive care or acute care units, whether they work night or day shift, years working at the hospital, and years since they graduated from their professional education program.

### Primary Outcome Measures

#### Feasibility of Participant Retention in Research

Feasibility of participant retention in research was indicated by ≥80% completion of follow-up surveys and role-plays at each time point. The study team administratively tracked the dates of completion to observe the length of time to completion of research activities.

#### Training Engagement and Completion

##### Self-Report of Training Completion

Nurses self-reported the percentage of completion (0-100) of 4 training activities on the posttraining survey, including (1) the didactic and demonstration portion, (2) practice with Client Bot Emily, (3) the standardized patient role-play persona Natalie for which they received automated feedback, and (4) review of this automated feedback for the persona Natalie via Lyssn Advisor.

##### Self-Report of Additional Use of Training Materials and Technologies

Nurses self-reported on the 6-month survey whether they reviewed, revisited, or engaged in additional practice with training activities after completing their posttraining survey (the date of which was provided to them). Training activities included the (1) didactic and demonstration material, (2) Client Bot Emily, and (3) reviewing their role-play on Lyssn Advisor.

##### Administrative and Lyssn.io Records of Training Completion

The study team tracked the dates of completion for all training activities via the eLearning training platform deployed using the REDCap, which was based on the nurses’ self-report on surveys as well as the standardized patient actor documentation of role-play completion. The Lyssn.io platform provides analytics on dates that users engage with Client Bot Emily and dates that users upload videos of role-plays for automated coding through Lyssn Advisor. The research team uploaded the videos on behalf of nurses; therefore, data on whether nurses accessed the Lyssn Advisor was not available.

#### Acceptability of the Training

##### SUS for Training Technologies

Immediately after interacting with Client Bot Emily and Lyssn Advisor, nurses completed the SUS [60]. The SUS has 10 statements responded to on a Likert-type scale ranging from 0=strongly disagree to 4=strongly agree. The scores range from 0 to 100, with higher scores indicating greater acceptability, with a cutoff of 68 out of 100. The SUS has demonstrated good internal consistency, reliability, and concurrent validity with other usability measures [62]. SUS internal consistency reliability was high for Client Bot Emily (Cronbach α=0.91; 16/17, 94%) and Lyssn Advisor (Cronbach α=0.89, 10/17, 59%).

##### Satisfaction With the Training

At the posttraining and 6-month follow-up surveys, nurses were asked how satisfied they were, on a scale of 0=not at all, 1=a little, 2=somewhat, 3=mostly, and 4=completely, with the following 4 aspects of the eLearning training in suicide safety planning: (1) the didactic content with demonstration of suicide safety planning, (2) interaction with Client Bot Emily, (3) role-play practice of suicide safety planning with a patient actor, and (4) review of their role-play and automated feedback from the Lyssn Advisor system.

At the posttraining and 6-month follow-up surveys, nurses were asked how well the eLearning training prepared them to do the following on a scale from 0=not at all, 1=a little, 2=somewhat, 3=mostly, and 4=completely: (1) do a suicide safety planning role-play with a patient actor; (2) engage patients on their unit in suicide safety planning; (3) talk with patients on their unit about suicidal thoughts, even if not doing suicide safety planning with them; (4) talk with patients on their unit about the patient’s experience of past suicide attempts or suicidal behaviors, even if not doing suicide safety planning with them; (5) talk with patients on their unit about resources for support or help with suicidal thoughts or urges; and (6) talk with patients on their unit about reducing access to lethal means.

### Secondary Outcome Measures

#### Quality of General Counseling and Suicide Safety Planning Skills

##### Standardized Patient Role-Plays

The study used 3 standardized patient role-plays [63] for evaluation purposes: postdidactic (Emelia persona), posttraining (Maureen persona), and at the 6-month posttraining follow-up (Brianna Persona) to observe the quality of nurses’ general counseling skills and suicide safety planning skills over time (Figure 1; Multimedia Appendix 1). An additional role-play served as a training activity only (Natalie persona).

The first author (DD) and study research coordinator (AP) created the role-play personas to reflect diversity of patient age, reason for hospitalization, suicide risk screening outcome, reasons for or drivers of their suicidality, and potential safety plan content (Multimedia Appendix 1). Each persona was a woman to align with the identity of the patient actor. An effort was made to limit the complexity and difficulty of the personas so that nurses would have the greatest opportunity to demonstrate their use of safety planning skills. For instance, each persona was able and willing to engage with the nurse in safety planning and had a response to any question the nurse might ask. Responses for each step of the safety planning process were identified ahead of time so the patient actor’s responses to nurses’ questions would remain standardized across nurses.

The research coordinator trained to be the standardized patient actor (AP) has a master’s degree in adult education and worked with research teams studying suicide prevention and treatment for 10 years prior. The training included (1) reading about theories of suicidality and discussing these with the first author (DD) and completing the didactic and demonstration portion of the eLearning training, (2) learning about standardized patient methodology by reading about this methodology [63] and discussing it with the first author, and (3) practicing as the patient actor for each role-play persona with the first author and receiving feedback on this performance. The first author and patient actor created a protocol to ensure standardization of the role-plays that included a description of each persona and responses to make to any nurse questions. The patient actor was trained to offer content for the safety plan only when the nurse asked for that content. For example, if the nurse did not ask the patient actor about whether they had access to lethal means, the patient actor did not offer this information to the nurse.

For each role-play, nurses signed up for a 30-minute session with the patient actor using web-based scheduling software. Varying days and times were offered for the role-plays and could be negotiated with the patient actor to find the best times for nurses. Nurses were provided with information about what to expect during the role-play, a brief description of the persona (Multimedia Appendix 1), and a reminder that they can review the didactic materials before completion. The role-play was conducted by videoconferencing software and recorded. Nurses completed the written safety plan with the patient actor electronically using screen-share or on a downloaded hard copy.

##### Quality of General Counseling Skills

The postdidactic, posttraining, and 6-month follow-up standardized patient role-plays were uploaded to and scored by Lyssn Advisor for the general counseling skills of empathy and collaboration, as defined by the Motivational Interviewing Treatment Integrity Scale 3.1.1 [61]. Empathy refers to how well the nurse demonstrated understanding of the patient’s thoughts and feelings and collaboration refers to how skillfully the nurse fostered power-sharing that allows the patient’s ideas to influence the direction of the session. Skills on each were rated on a scale from 0=low to 5=high with a cutoff of 3.5 for what may be considered basic ability and 4 for advanced ability.

##### Quality of Suicide Safety Planning Skills

The first author (DD) rated the quality of nurse safety planning by rating nurse performance on standardized patient role-plays using the Safety Plan Intervention Rating Scale (SPIRS) Version 3 (GK Brown, unpublished data, January 2020). The SPIRS is a rating scale created by the developers of the Safety Planning Intervention to evaluate whether various aspects of safety planning are completed and how well they are completed on a scale with 0=not present, 1=inadequate, 2=satisfactory, and 3=excellent. The rating scale assesses 6 general aspects of safety planning, such as asking the patient for a narrative of a recent suicidal crisis and providing a rationale for completing a safety plan, and 6 aspects corresponding to the steps outlined on the written safety plan. For each step of the plan, criteria include whether nurses generated specific and individualized content for that step, provided a rationale for the step, and asked for feedback (eg, usefulness, helpfulness, and troubleshooting barriers) on the ideas generated for the step. Each of the 2 subscales contains 6 items which are summed. Scores on each subscale range from 0 to 18; a cutoff of 14 indicates adequate safety planning quality for that subscale. The first author was trained by the developers to reliably rate role-plays using the SPIRS and subsequently rated the postdidactic, posttraining, and 6-month follow-up role-plays.

#### Knowledge and Confidence in Delivering Suicide Safety Planning

On each study survey, nurses reported their perceived knowledge of and confidence to deliver suicide safety planning on 2 measures created for this study based on aspects of the Safety Planning Intervention (Multimedia Appendix 1). Nurses were asked to report on 32 aspects of doing safety planning with regard to (1) how knowledgeable they felt about each aspect and (2) how confident they were doing each in a role-play with a standardized patient. Nurses rated their knowledge and confidence on a Likert-type scale from with 0=not at all, 1=a little, 2=somewhat, 3=a good bit, and 4=very. Items were summed and divided by the total number of items completed to obtain a score between 0 to 4; higher scores indicated greater self-perception of knowledge and confidence in suicide safety planning. Internal consistency reliability was high for both measures with estimates of Cronbach α between 0.91 and 0.97 for both measures across time points.

#### Intention to Deliver Suicide Safety Planning

On each study survey, nurses rated how much they agree that they intend to engage patients in suicide safety planning on their unit after being trained in suicide safety planning on a scale with 0=do not agree at all, 1=agree a little, 2=agree somewhat, 3=agree quite a bit, and 4=completely agree; nurses could indicate that they do not know.

#### Changes in Behavior With Patients at Risk of Suicide

Nurses were asked on the posttraining and 6-month follow-up surveys if they had done any of the following because of their participation in the eLearning training (not something they would have done otherwise): (1) engage patients on their unit in suicide safety planning, (2) talk with patients on their unit about reducing access to lethal means (outside of doing a full safety plan), (3) talk more with patients on their unit about suicidal thoughts than they previously have, (4) talk more with patients on their unit about patients’ experience of past suicide attempts or suicidal behaviors than they previously have, (5) talk more with patients on their unit about resources for support or help with suicidal thoughts or urges than they previously have, and (6) change the way they interact with suicidal patients in some other way.

### Plan of Analysis

We examined descriptive statistics for all outcomes, including frequencies for categorical variables and the mean and SD for continuous variables. We observed means and CIs based on the SEM for continuous variables observed across time points. As a supplementary analysis, we descriptively explored the association between the length of time nurses took to complete each role-play and suicide safety planning quality per the SPIRS for the postdidactic, posttraining, and 6-month follow-up role-plays. There were very little missing data for completed surveys (<1%).

## Results

### Participants

A total of 19 nurses enrolled in the study; 18 (95%) participated. Of these 18 nurses, the majority identified as female (n=17, 94%) and White (n=13, 72%; Table 1). All worked on units that treat traumatically injured patients, the majority worked on acute care units during day shifts (12/18, 67%), and nurses had been working at the study hospital an average of 5.4 (SD 4.6) years.

**Table 1:** Demographic characteristics of hospital nurses enrolled in the formative evaluation.

			Values^a^
**Gender, n (%)**			
	Women	17 (94)	
	Men	1 (6)	
**Racial and ethnic identity, n (%)**			
	Asian	3 (17)	
	White	13 (72)	
	Hispanic or Latino, White	1 (6)	
	Declined to answer	1 (6)	
**Type of unit, n (%)**			
	Acute care	16 (89)	
	Intensive care	2 (11)	
**Work shift, n (%)**			
	Day	12 (67)	
	Night	6 (33)	
Age (y), mean (SD)			31.4 (6.0)
Years since graduation from professional education program, mean (SD)			6.2 (4.9)
Years working at the study hospital, mean (SD)			5.4 (4.6)

^a^Percentages based on 18 nurses who consented to the study and completed a demographics questionnaire. All nurses worked on units serving traumatically injured patients.

### Primary Outcomes

#### Feasibility of Participant Retention in Research

**Figure 2:**
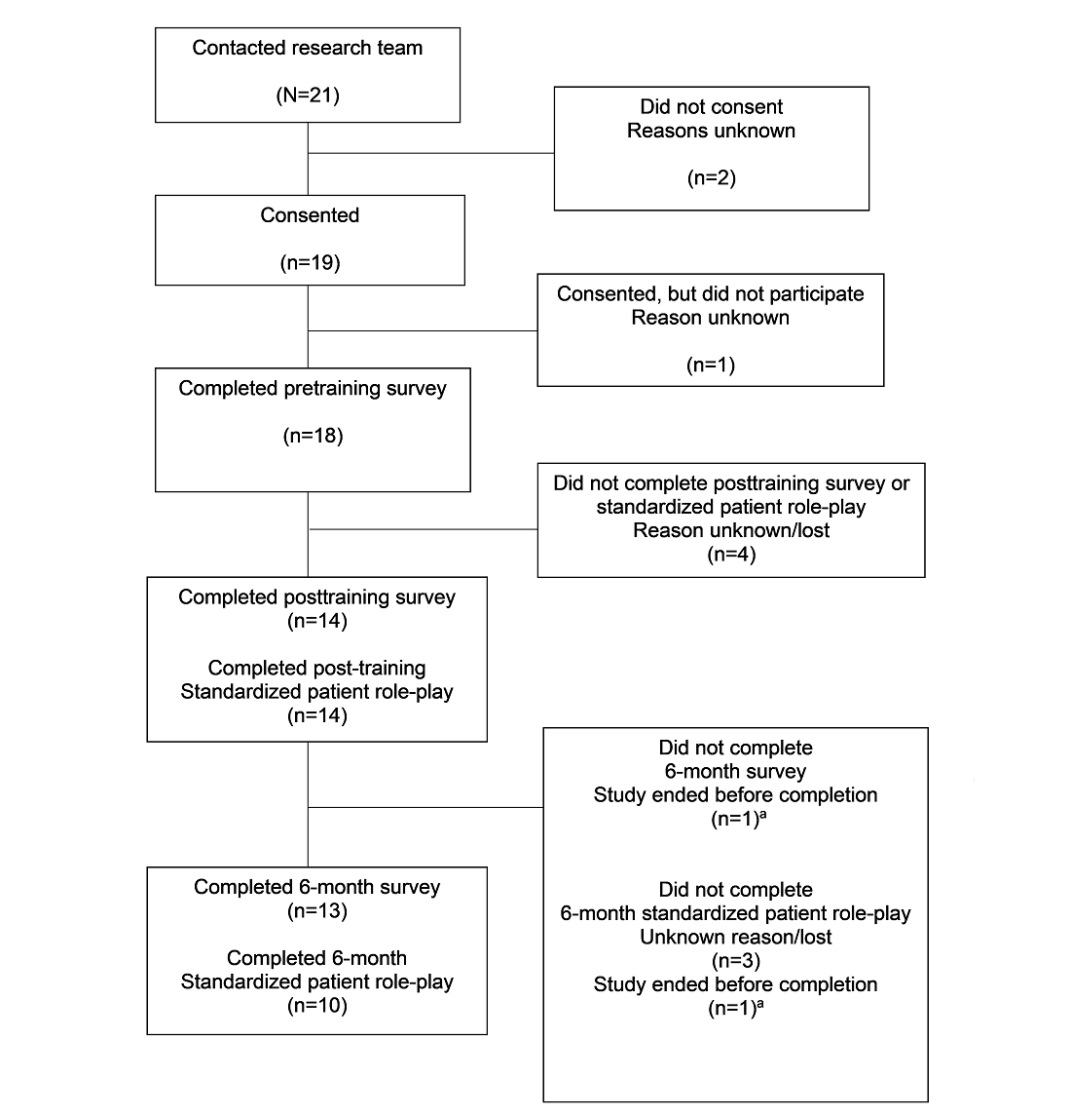
Nurse completion of research surveys and standardized patient role-plays over the course of a formative evaluation of a training in suicide safety planning. The study recruited nurses working with medically hospitalized patients. Recruitment occurred between November 1, 2021, and May 31, 2022. Nurses were invited to complete surveys before, after, and 6 months following the training. Nurses were invited to complete 30-minute standardized patient role-plays to assess the quality of nurses’ general counseling and safety planning skills. Role-plays were completed (1) after learning about safety planning and watching a demonstration of how to do it, (2) after practicing general counseling microskills with the simulated patient Client Bot Emily, and (3) after viewing their automated scores for the quality of their general counseling skills via Lyssn Advisor. ^a^The study ended 3 months after one of the nurses completed the posttraining survey and standardized patient, and therefore the nurse was not able to complete the 6 month follow-up survey or 6 month follow-up standardized patient.

Of the 19 nurses who consented to participate in the study, 18 (95%) completed the pretraining survey (Figure 2). Moreover, 14 (74%) completed both the posttraining survey and posttraining standardized patient role-play. Overall, 13 (68%) completed the 6-month follow-up survey and 10 (53%) completed a 6-month follow-up standardized patient role-play.

On average, the 74% (14/19) of nurses who completed the posttraining survey did so 16.5 (SD 18.4; minimum=0, maximum=50) days after completing the training and completed the posttraining standardized patient role-play 30.4 (SD 18.1; minimum=2, maximum=63) days after completing the training. On average, the 13 (68%) out of 19 nurses who completed the 6-month follow-up survey did so 199.6 (SD 38.6; minimum=139, maximum=301) days after they completed the training and, on average, the 13 (68%) out of 19 nurses who completed the 6-month follow-up standardized patient role-play did so 202.1 (SD 38.9; minimum=134, maximum=265) days after completing the training.

#### Training Engagement and Completion

##### Self-Report of Training Completion

All 14 nurses who completed the posttraining survey reported completing at least 50% of each activity (didactic and demonstration portion: mean 94%, SD 14%; Client Bot Emily: mean 82%, SD 18%; role-play: mean 95%, SD 11%; Lyssn Advisor: mean 91%, SD 12%). Twelve (86%) out of the 14 nurses who completed the training reported completing 90% to 100% of the didactic and demonstration portion, 43% (6/14) reported completing 90% to 100% of the practice with feedback with Client Bot Emily activity, 86% (12/14) reported completing 90% to 100% of a role-play with a standardized patient, and 64% (9/14) reported completing 90% to 100% of the Lyssn Advisor feedback activity.

##### Self-Report of Additional Use of Training Materials and Technologies

Of the 13 nurses who completed the 6-month follow-up survey, 9 (69%) reported reviewing the didactic and demonstration material, 3 (23%) reported returning to complete additional practice with Client Bot Emily, and 6 (46%) returned to review their role-play and scores on Lyssn Advisor after completing the initial training.

##### Administrative and Lyssn.io Records of Training Completion

**Figure 3:**
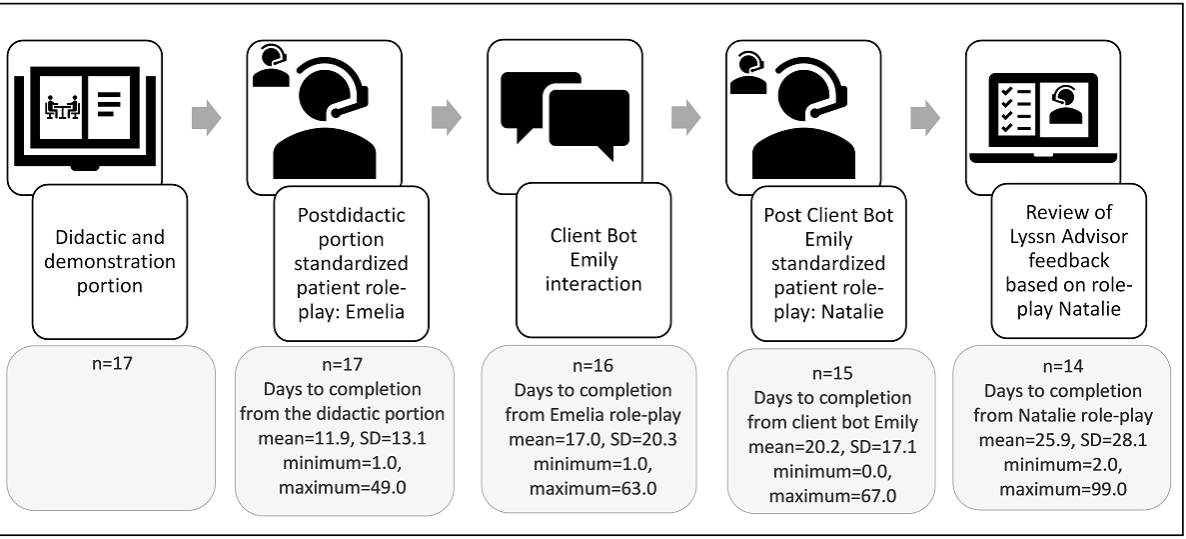
Completion rates for the training in suicide safety planning and the number of days between training activities for the 18 nurse participants in the formative evaluation of the training. Participants were nurses who work with medically hospitalized patients. Data generated upon completion of activities by the nurses were documented in the REDCap (Research Electronic Data Capture) web-based survey system and study team administrative records. For the 14 nurses who completed all training activities, the average number of days between start and completion of all training activities was 76.9 (SD 45.8; minimum=27.0, maximum=212.0).

On the basis of survey completion rates and the research team’s administrative records, 17 (89%) of the 19 nurses who consented to participate began the training and 14 (82%) of the 17 who began the training completed it (Figure 3). On average, it took these 14 nurses 76.9 days (SD 45.8) to complete all training activities. Per Lyssn.io Client Bot Emily analytics, 16 (94%) of the 17 nurses who began the training, interacted at least once with Client Bot Emily: for 11 (69%) of the 16 nurses who interacted with Client Bot Emily, they interacted on 1 date; 4 (25%) of the 16 interacted on 2 different dates, which were both within the training period; and 1 (6%) of the 16 interacted on 3 different dates, also all within the training period (ie, before completing the posttraining survey).

#### Acceptability of the Training

##### SUS for Training Technologies

On average, the SUS score for the 16 (94%) out of the 17 nurses who began the training and who completed the Client Bot Emily activity was 70.3 (SD 19.7) and for the 14 (82%) out of the 17 nurses who began the training and who completed the Lyssn Advisor feedback activity was 65.4 (SD 16.3).

##### Satisfaction With the Training

Descriptively, the highest satisfaction score observed was for the standardized patient role-play practice and the lowest was for Client Bot Emily, which held for both the posttraining and the 6-month follow-up surveys (Table 2). To better understand the lower satisfaction scores for Client Bot Emily, we reviewed nurse comments about their experience with the technology they could make at the end of the SUS survey. Eight nurses mentioned that a challenge with Client Bot Emily was that she did not consistently respond coherently to the nurses’ statements and frustration that Client Bot Emily was not able to maintain a meaningful conversation. For Lyssn Advisor, there was only 1 comment about any challenges with the platform; 1 nurse noted it was *not very user friendly*.

**Table 2:** Nurses’ self-reported satisfaction with training components and self-reported preparedness for various suicide prevention activities, based on posttraining and 6-month follow-up survey responses^a^.

			Posttraining (n=14), mean (SD; 95% CI^b^)		6-month follow-up (n=13), mean (SD; 95% CI^b^)
**Satisfaction**					
	Didactic and demonstration portion	3.1 (0.8; 2.6-3.5)		3.2 (0.8; 2.7-3.6)	
	Client Bot Emily	1.1 (1.1; 0.5-1.6)		1.5 (1.2; 0.8-2.1)	
	Role-play practice	3.5 (0.5; 3.2-3.8)		3.5 (0.5; 3.3-3.8)	
	Lyssn Advisor feedback	2.5 (0.6; 2.0-3.0)		2.6 (1.0; 2.1-3.1)	
**Preparation^c^**					
	Doing a role-play	3.0 (0.9; 2.5-3.5)		3.2 (0.7; 2.8-3.5)	
	Safety planning with patients	2.5 (1.0; 1.9-3.0)		2.9 (1.0; 2.4-3.5)	
	Talking with patients about suicidality	2.7 (0.8; 2.3-3.1)		3.3 (0.7; 3.0-3.7)	
	Talking with patients about past suicide behavior	2.5 (0.9; 2.1-3.0)		3.1 (0.9; 2.6-3.6)	
	Talking with patients about resources for suicidality	2.2 (0.9; 1.7-2.7)		2.8 (0.8; 2.3-3.2)	
	Talking with patients about lethal means	2.2 (1.1; 1.6-3.3)		2.8 (1.1; 2.1-3.4)	

^a^Satisfaction with each activity rated on a 0 to 4 scale, with higher scores indicating greater satisfaction. Preparation indicates nurses’ self-report of how well the training prepared them for each activity, rated on a 0 to 4 scale with higher scores indicating better preparedness.

^b^95% CIs presented are based on the SEM.

^c^Sample size was 13 (93%) out of the 14 who completed the posttraining survey and 12 (92%) out of the 13 who completed the 6-month follow-up survey for all preparation items except doing a role-play, for which 1 nurse marked don’t know.

Descriptively, on the posttraining survey, the highest score observed for how well the eLearning training prepared nurses to engage in suicide care activities was for doing a suicide safety planning role-play with a patient actor, whereas the highest score at the 6-month follow-up was for talking with patients about suicidal thoughts (Table 2). The lowest scores at both time points were the same for talking with patients about resources for support or help with suicidal thoughts and talking with patients about reducing access to lethal means.

### Secondary Outcome Measures

#### Quality of General Counseling and Suicide Safety Planning Skills

**Figure 4:**
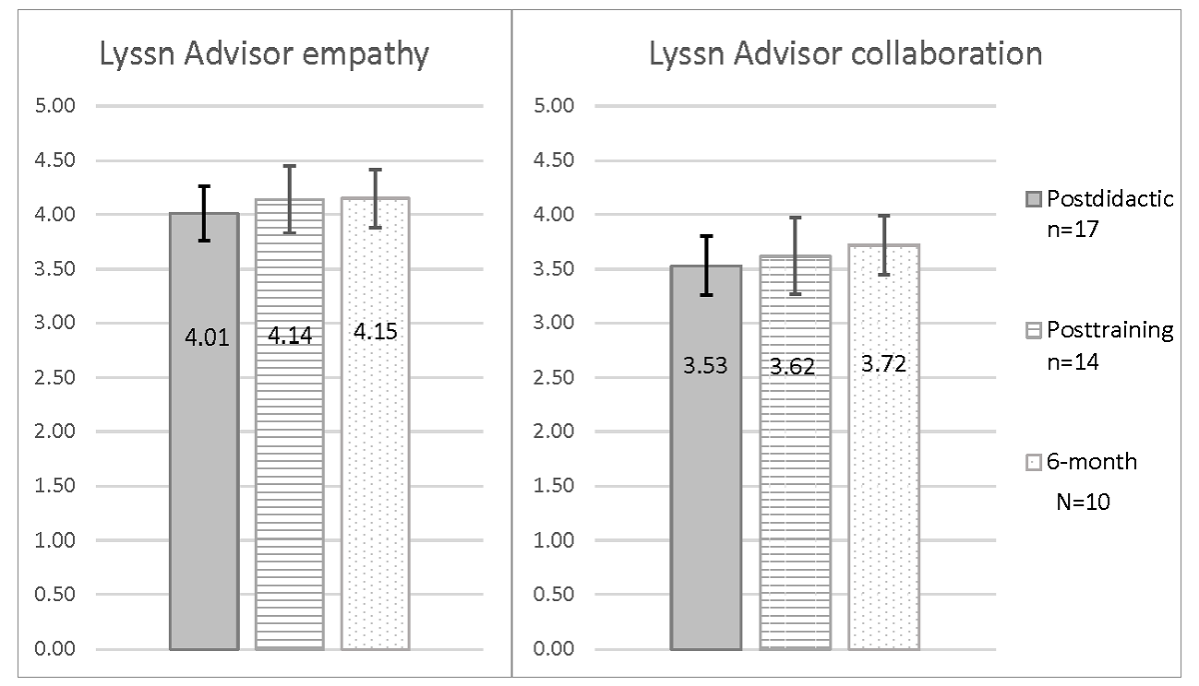
Lyssn Advisor mean scores for empathy and collaboration based on suicide safety planning standardized patient role-plays completed by nurse participants as part of the formative evaluation. Participants were nurses working with medically hospitalized patients. Scores for standardized patient role-plays presented are those completed after (1) the didactic portion of the training in which nurses learned about safety planning and watched demonstration videos of safety planning, (2) practicing general counseling microskills with the simulated patient Client Bot Emily, and (3) viewing automated coding scores for general counseling skills through Lyssn Advisor (posttraining). Empathy refers to how well the nurse demonstrated understanding of the patient’s thoughts and feelings and collaboration refers to how skillfully the nurse fostered power-sharing that allows the patient’s ideas to influence the direction of the session. Scores ranging from 0 to 5, with a cutoff of 3.5, may be considered basic ability and 4 as advanced ability. Error bars represent 95% CIs based on the SEM.

**Figure 5:**
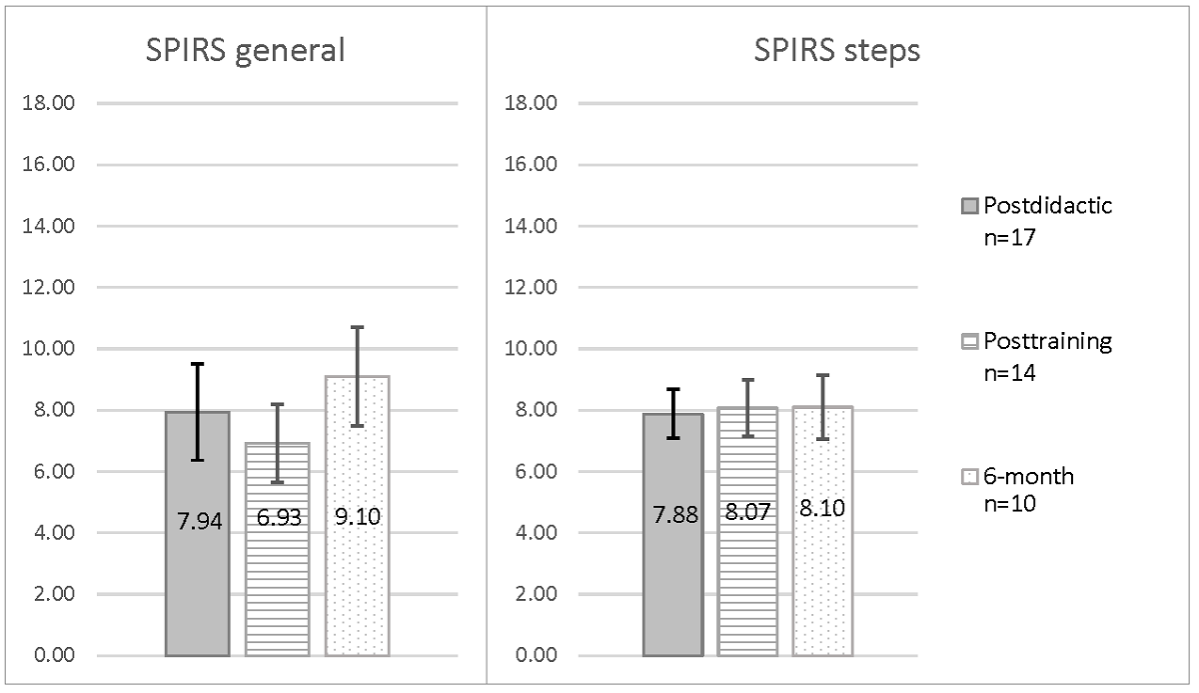
Mean scores on the Safety Plan Intervention Rating Scale (SPIRS) based on suicide safety planning standardized patient role-plays completed by nurse participants as part of the formative evaluation. Participants are nurses working with medically hospitalized patients. Scores for standardized patient role-plays presented are those completed after (1) the didactic portion of the training in which nurses learned about safety planning and watched demonstration videos of safety planning, (2) practicing general counseling microskills with the simulated patient Client Bot Emily, and (3) viewing automated coding scores for general counseling skills through Lyssn Advisor (posttraining). Role-plays were rated on the SPIRS by the first author (DD) by viewing video recordings of each role-play. The SPIRS includes scores for both general aspects of safety planning as well as for each of the safety planning steps. Scores ranged from 0 to 18, with cutoff scores of 14 or greater indicating high-quality safety planning. Error bars represent 95% CIs based on the SEM.

Role-play completion rates, average length of time for each role-play, and role-play quality outcomes are presented in Table 3. Descriptively, nurses’ average scores according to Lyssn Advisor for general counseling skills were at or above what is considered basic ability and at or above what is considered advanced ability for empathy (Figure 4). Descriptively, nurses’ scores on average across both the SPIRS general safety planning subscale and subscale for safety planning steps were below the cutoff for adequate safety planning quality and remained at similar levels over time (Figure 5). Only 1 nurse attained an SPIRS score at or above the cutoff (≥14), which occurred for the SPIRS general subscale on the postdidactic role-play.

**Table 3:** Descriptive statistics of the quality of general counseling and suicide safety planning skills based on nurses’ performance on 30-minute role-plays with a patient actor rated using the automated coding system Lyssn Advisor human coding with the Safety Plan Intervention Rating Scale, Version 3 (SPIRS)^a^.

	Values, n (%)^b^	Role-play length, mean (SD)	Lyssn Advisor empathy, mean (SD; 95% CI)^c^	Lyssn Advisor collaboration, mean (SD; 95% CI)^c^	SPIRS general^d^, mean (SD; 95% CI)^c^	SPIRS steps, mean (SD; 95% CI)^c^
Postdidactic	17 (89)	24.51 (6.20)	4.01 (0.37; 3.76-4.26)	3.53 (0.59; 3.26-3.80)	7.94 (3.29; 6.37-9.51)	7.88 (1.69; 7.08-8.68)
Posttraining	14 (74)	28.81 (7.20)	4.14 (0.62; 3.83-4.45)	3.62 (0.66; 3.27-3.97)	6.93 (2.43; 5.66-8.20)	8.07 (1.77; 7.15-8.99)
6-months follow-up	10 (53)	29.00 (4.32)	4.15 (0.43; 3.88-4.42)	3.72 (0.45; 3.45-3.99)	9.10 (2.60; 7.49-10.71)	8.10 (1.66; 7.06-9.14)

^a^Role-plays were completed after (1) the didactic portion of the training in which nurses learned about safety planning and watched demonstration videos of safety planning, (2) practicing general counseling microskills with the simulated patient Client Bot Emily, and (3) viewing automated coding scores for general counseling skills through Lyssn Advisor.

^b^Percentages are out of 19 nurses who provided consent.

^c^95% CIs presented are based on the SEM.

^d^SPIRS general includes the sum of 6 items of general skills for the delivering suicide safety planning and SPIRS steps includes the sum of 6 items of completing the safety planning steps. Both scales are rated from 0 to 3 with higher scores indicating higher quality of skills. Lyssn Advisor empathy and collaboration scores are on a scale from 0 to 5; higher scores indicate higher quality skills and cutoff of 3.5 for what may be considered basic ability and 4 for advanced ability.

We reviewed nurses’ posttraining role-play scores (n=14) to explore areas of strength and weaknesses among the general and specific aspects of safety planning skills. Among those nurses who completed the training and a posttraining role-play within the SPIRS general safety planning subscale, the most frequently low scoring items reflected inadequately or not asking the patient actor to provide a narrative of a recent suicidal crisis and aligning this narrative to the risk curve of suicidal crises (13/14, 93%); the most frequently high scoring item reflected satisfactory or excellent collaborative engagement with the patient actor in the safety planning process (12/14, 86%). Among the items for safety planning steps, low scores were commonly due to nurses not providing a complete rationale or not asking for feedback or troubleshooting with the patient actor for each step. On average across all 6 steps, 75% (SD 11%) of nurses did not provide a rationale for the steps and 94% (SD 5%) did not ask for feedback (eg, helpfulness or usefulness, troubleshooting barriers). Nurses frequently, however, met criteria for identifying specific and individualized elements of the plan, with an average of 95% (SD 6%) meeting this criterion across the 6 steps.

Exploratory descriptive analyses of the length of time nurses took to complete the role-play and the quality of the suicide safety planning intervention as scored using the SPIRS, including scatterplots and bivariate correlations for the postdidactic, posttraining, and 6-month follow-up role-plays are presented in Multimedia Appendix 1. We observed a high, positive correlation between length of time and the SPIRS scores at the postdidactic role-play, which also had the largest sample size.

#### Knowledge and Confidence in Delivering Suicide Safety Planning

**Figure 6:**
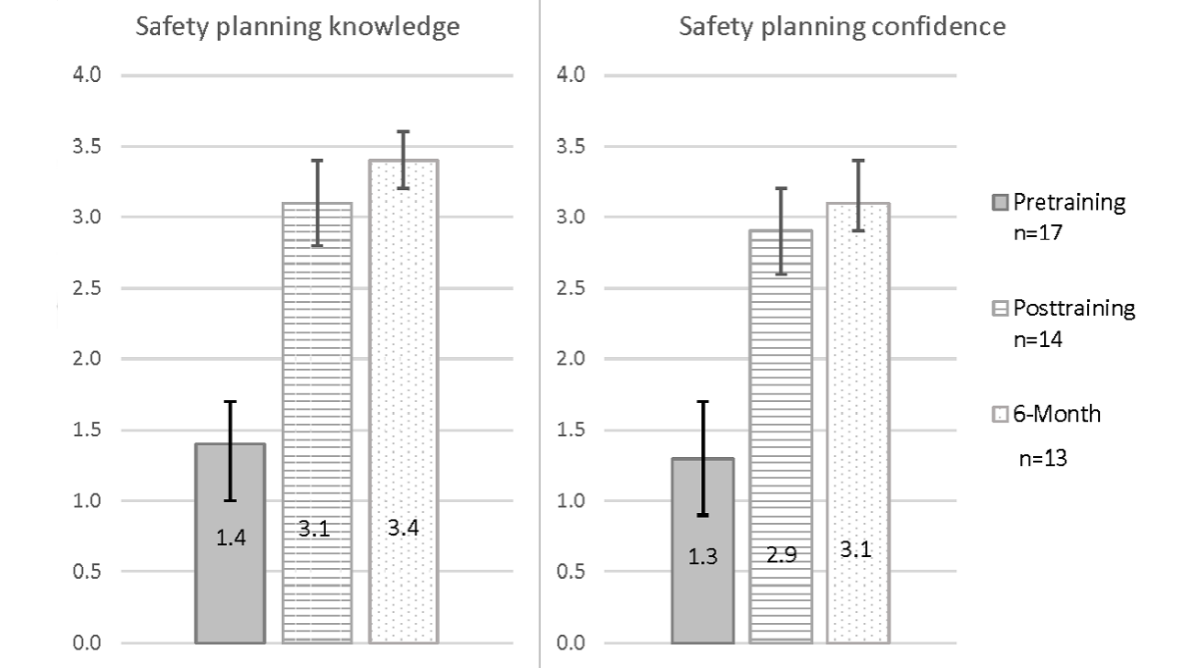
Mean scores for self-reported knowledge and confidence in delivering suicide safety planning based on nurses’ self-report on surveys completed as part of the formative evaluation of the training in suicide safety planning. Participants were nurses working with medically hospitalized patients. On the pretraining, posttraining, and 6-month follow-up surveys, nurses were asked to report on 32 aspects of doing safety planning with regard to (1) how knowledgeable they felt about each aspect and (2) how confident they were doing each in a role-play with a standardized patient. Nurses rated their knowledge and confidence on a Likert-type scale from with 0=not at all, 1=a little, 2=somewhat, 3=a good bit, and 4=very. Items were summed and divided by the total number of items completed to obtain a score between 0 to 4; higher scores indicated greater self-perception of knowledge and confidence in suicide safety planning. Error bars represent 95% CIs based on the SEM.

Descriptively, nurses’ scores on both the measures of self-perceived knowledge of and confidence in delivering suicide safety planning were higher at the posttraining and 6-month follow-up surveys than they were at the pretraining survey (Figure 6; Table 4).

**Table 4:** Descriptive statistics for nurses’ self-reported knowledge and confidence in suicide safety planning based on survey responses over timea.

	Pretraining (n=18), mean (SD; 95% CI^b^)	Posttraining (n=14), mean (SD; 95% CI^b^)	6-months follow-up (n=13), mean (SD; 95% CI^b^)
Knowledge	1.4 (0.7; 1.0-1.7)	3.1 (0.5; 2.8-3.4)	3.4 (0.4; 3.2-3.6)
Confidence	1.3 (0.8; 0.9-1.7)	2.9 (0.5; 2.6-3.2)	3.1 (0.4; 2.9-3.4)

^a^Nurses were asked to report on 32 aspects of doing safety planning with regards to 1) how knowledgeable they felt about each aspect and 2) how confident they were doing each in a role-play with a standardized patient. Nurses rated their knowledge and confidence on a Likert-type scale from with 0=not at all, 1=a little, 2=somewhat, 3=a good bit, and 4=very. Items were summed and divided by the total number of items completed to obtain a score between 0-4; higher scores indicated greater self-perception of knowledge and confidence in suicide safety planning.

^b^95% CI values are based on the SEM.

#### Intention to Deliver Suicide Safety Planning

Descriptively, nurses’ scores on the 1-item measure of intention to engage patients in suicide safety planning on their unit after being trained in suicide safety planning was higher on the pretraining survey than the posttraining and 6-month follow-up surveys. At the pretraining survey, 1 nurse reported they did not know if they would do safety planning with patients; among the other 94% (17/18) nurses, the mean was 3.2 (SD 0.1; 95% CI 3.0-3.4). At the posttraining survey, 14% (2/14) nurses reported they did not know if they would; among the other 86% (12/14) nurses, the mean was 2.3 (SD 0.3; 95% CI 1.7-2.8). At the 6-month follow-up survey, 15% (2/13) nurses reported they did not know if they would; among the other 85% (11/13) nurses, the mean was 2.4 (SD 0.2; 95% CI 1.9-2.8).

#### Changes in Behavior With Patients at Risk of Suicide

At the posttraining survey, 43% (6/14) nurses reported they did not know if they had made any change in behavior or skipped the item; the remaining 57% (8/14) reported they had made at least one behavioral change because of the training (Table 5). At the 6-month follow-up survey, 23% (3/13) nurses reported they did not know if they had made any change in behavior or skipped the item; the remaining 77% (10/13) reported they had made at least one behavioral change. The most common behavioral change was *Talk more with patients on my unit about suicidal thoughts than I previously have*. The least common behavioral change was, *Engage patients on my unit in suicide safety planning*.

**Table 5:** Frequencies for nurses’ self-report of making behavioral changes in working with at-risk patients because of the training based on posttraining and 6-month follow-up survey responses^a^.

	Posttraining (n=14), n (%)	6-months follow-up (n=13), n (%)
Engage patients on my unit in suicide safety planning	1 (7)	3 (23)
Talk with patients on my unit about reducing access to lethal means (outside of doing a full safety plan)	2 (14)	4 (31)
Talk more with patients on my unit about suicidal thoughts than I previously have	6 (43)	7 (54)
Talk more with patients on my unit about their experience of past suicide attempts or suicidal behaviors than I previously have	5 (34)	5 (38)
Talk more with patients on my unit about resources for support or help with suicidal thoughts or urges than I previously have	3 (21)	7 (54)
Change the way I interact with suicidal patients in some other way^b^	1 (7)	4 (31)
Do not know or skip	6 (43)	3 (23)

^a^Nurses indicated whether they had made each of the listed changes. Nurses could indicate that they either do not know if they made the change in their behavior with patients or could opt to skip the question.

^b^Two nurses specified on some other way: (1) Be less fearful to ask questions about past suicidal behavior, and (2) being less timid to talk with patients about suicide and how they are feeling.

## Discussion

### Principal Findings: Feasibility and Acceptability of the eLearning Training

Developing feasible and acceptable continuing education training in suicide prevention with opportunities for skills practice and feedback on skill performance is critical for integrating suicide prevention into general medical settings. Findings from this formative evaluation of an eLearning training in suicide safety planning with acute and intensive care nurses indicate that completion of the training activities and use of novel technologies within this context are feasible. Specifically, of those nurses who started the training, 82% (14/17) completed it. However, on average, it took 2.5 months (mean 76.9, SD 45.8 days) for nurses to complete all training activities despite the research team’s expectation that it would take no more than 1 month. It may be that it is difficult for nurses to find the time to complete the training activities as volunteers in a research study. Although the training was designed so that it could be flexibly completed in parts, it may be a more feasible model for the training to be completed during work hours and nurses given time away from clinical duties to complete the training.

Acceptability of and satisfaction with the novel technologies varied and indicates some areas for improvement in the technology. Nurses rated Client Bot Emily above the cutoff for usability (mean 70.3, SD 19.7) but only a little satisfying (mean 1.1, SD 1.1), with the reason given by nurses that the virtual patient was not able to have consistently meaningful responses to the nurses’ comments and their attempts to practice open-ended questions and reflective statements. On average, nurses rated the Lyssn Advisor system as under the cutoff for usability (mean 65.4, SD 16.3) but more than somewhat satisfying (mean 2.5, SD 0.6). It may be the Lyssn Advisor platform is complex enough that nurses needed a brief tutorial on the features and how to interact with it; nurses were only provided instructions for how to access the system and what to scores to review. With regard to Client Bot Emily, the ability for artificial intelligence to generate ongoing and meaningful conversation is a growing area of work and will likely improve with advances in methodology and computing capacity [64]. Nurses may find a future version of a virtual patient like Client Bot Emily with increased conversational capacity useful for practicing counseling skills.

Nurses were satisfied with other aspects of the training, endorsing on average mostly satisfied with the didactic and demonstration activities (mean 3.1, SD 0.8) and greater than mostly satisfied with the role-play with a standardized patient actor (mean 3.5, SD 0.5). Role-play is a critical method for skills-based training and increases satisfaction with trainings [28,32,65], but it can increase the cost, given the need to pay patient actors, and can be challenging to implement in routine continuing education settings. An alternative to role-play with a human patient actor is to have trainees role-play with other trainees. This method appears to be more cost-effective [66] and can be as effective on learning outcomes and even incur additional benefits, such as greater patient empathy resulting from taking on the role of the patient [67]. Standardized patient actors may still be more appropriate when trainee capability needs to be measured with greater accuracy, such as in formal educational environments, to evaluate student competency.

Findings regarding nurses’ perception of how well the training prepared them to engage in various activities, indicate room for improvement on aspects of safety planning as well as talking to patients about suicidality in general. Scores on all the activities we inquired about were descriptively in the *somewhat* to *mostly* range with the lowest scores on identifying local resources and lethal means counseling. These scores were likely the lowest because the training did not have content on local resources nurses could offer to patients and had limited depth on the types of strategies to consider for helping patients limit access to lethal means. Both didactic content in these areas as well as feedback on specific safety planning skills in addition to feedback on general counseling skills are needed to help nurses feel more prepared to do safety planning.

### Secondary Findings: Learning Outcomes

Examination of learning outcomes suggests the eLearning training is promising with regard to improvement in nurses’ knowledge and confidence in delivering suicide safety planning with patients. On average, nurses endorsed a good bit of knowledge (mean 3.1, SD 0.5) and confidence (mean 2.9, SD 0.5) after the training and endorsed a little bit for knowledge (mean 1.4, SD 0.7) and confidence (mean 1.3, SD 0.8) before the training. This is an encouraging finding because both perceiving oneself as knowledgeable about and capable of delivering an evidence-based intervention like suicide safety planning are known to influence the motivation of health care workers to deliver it [68]. However, there are numerous other personal, social, and environmental factors influencing delivery [69]. For instance, previous research with acute and intensive care nurses in the study hospital indicates that nurses often do not have the 30 to 45 minutes of uninterrupted time to complete safety planning with patients [19]. Practical challenges may explain why on average nurses endorsed that they agree somewhat that they intended to engage patients on their unit in suicide safety planning after completing the training (mean 2.3, SD 0.3) as well as why only 23% (3/13) of the nurses who completed the 6-month follow-up survey reported engaging patients on their unit in suicide safety planning during the study period. Despite the low use of suicide safety planning, an encouraging finding was that over half (8/13, 57%) of nurses at the 6-month follow-up reported that the eLearning training led them to change their behavior in positive ways with patients on their units at risk of suicide. The most common changes were *Talking more with patients on my unit about suicidal thoughts than I previously have* and *Talking more with patients on my unit about resources for support or help with suicidal thoughts or urges than I previously have*.

Although self-reported knowledge and confidence are frequent targets of training, having greater knowledge and confidence does not necessarily translate to high-quality intervention delivery when observed by a trained rater [70,71]. Consistent with such findings, nurses in this study endorsed a high degree of self-reported knowledge and confidence in skills for suicide safety planning after the training; however, on average, nurses did not receive ratings above the expert-derived cutoff for high-quality delivery of suicide safety planning on the SPIRS for the posttraining standardized patient role-plays. A review of the Lyssn Advisor and SPIRS scores suggests nurses did well with skills of collaboration, empathy, and identifying specific strategies for each of the steps of the safety plan. Areas in need of further training appear to be providing a rationale for safety planning overall and for each step of the plan as well as troubleshooting barriers to ensure the strategies identified in the plan are feasible. Providing a compelling rationale for an intervention and why it is expected to work for the condition being treated has been shown to increase patient engagement in psychotherapy [72,73]. Providing rationales and troubleshooting barriers to using skills are integral parts of cognitive-behavioral interventions, such as the suicide safety planning intervention [13] that aims to teach patients how, why, and when to use skills taught in their daily life [74]. Although the training materials and the video demonstration included examples of what to say for a rationale and what to ask to troubleshoot barriers, the importance of these elements could be more strongly emphasized. Furthermore, it is likely that nurses need tailored coaching from virtual patients and automated coding technologies to reinforce the importance of these elements, ensure that the rationales are accurate and understandable to the patient, and that barriers to using the safety plan are effectively addressed.

As a pilot research program using the existing metrics of general counseling skills (ie, collaboration and empathy) on the Lyssn Advisor system, this study did not include scores or feedback to nurses for skills specific to suicide safety planning, such as how well nurses did with providing a rationale for safety planning or the quality of strategies identified for the safety plan. Automated coding for suicide safety planning does not yet exist; however, automated coding for other types of cognitive-behavioral interventions is currently in development [75] and may translate to the safety planning context. Future iterations of this technology-enhanced training would likely benefit from feedback on giving rationales and troubleshooting barriers. A larger question remains as to what components of safety planning are critical for nurses to incorporate and deliver with high quality. Although higher quality safety plans and more collaborative safety planning have been shown to be associated with better patient outcomes [76,77], there is little know-how about the mechanisms of safety planning and the specific skills or components of the intervention associated with patient outcomes [78]. Future research to optimize suicide safety planning and its components, given the constrains of the acute care medical setting [79] would be helpful to identify which skills are critical to teach nurses.

Given the time constraints in the acute care context, we explored the association between the length of time nurses spent in the role-play with the patient actor and their SPIRS subscale scores. Although nurses were asked to spend 30 minutes in the role-play, the actual length of time spent varied. The general pattern appeared to be that longer role-plays were associated with higher suicide safety planning quality for the first role-play, but not subsequent role-plays. Such a pattern suggests that once the study nurses were more familiar with the role-play process or had some previous practice with a suicide safety planning role-play, the less the length of time mattered in the quality of their delivery. However, owing to the small and varying sample size over time, these findings must be interpreted with caution. In addition, these findings may only be relevant in this pilot-study context with the type of training nurses received, the suggested 30 minutes time-limit for the role-play, and the range of SPIRS scores observed.

### Implications for Refinements to the eLearning Training and Future Evaluation

Findings from this formative evaluation suggest that the eLearning continuing education model, harnessing novel technologies through web-based platforms as well as role-play with feedback, is a promising approach for efficiently training nurses in suicide safety planning. However, there is room for improvement to increase the acceptability, help nurses feel more prepared to safety plan with patients, and deliver the intervention with high quality. One modification to the technology to increase acceptability may be to increase the conversational capacity of the virtual patient. Another modification needed is to increase the training material emphasis on the specific suicide safety planning skills, such as providing patients with a rationale for safety planning and for each step of the plan as well as troubleshooting barriers to any strategies identified in the plan. Nurses may also need additional support in how to navigate the Lyssn Advisor platform. A final consideration is related to the implementation of the training. The months required to complete the 3- to 4-hour training and the time nurses took between activities suggests the training activities may have been challenging for nurses to complete as volunteers. It is possible that completion would be more feasible and reach more nurses if it could be tied to continuing education credits and meet requirements for state licensure [80]. Messaging from nursing and hospital leadership on the importance of suicide-prevention training and the importance of suicide care in the context of medical care could also encourage nurses’ engagement in training [81,82].

A primary consideration for a future rigorous evaluation is the ability to recruit and retain participants. The study aimed to recruit 20 nurse participants and retain 80% in the study over time. We recruited 19 nurses and 18 (95%) participated in the study. Of the 18, we retained 78% (n=14) at the posttraining survey and 72% (n=13) at the 6-month follow-up. Retention in the 6-month standardized patient role-play was 55% (n=10). These retention rates were below feasibility cutoffs for a future study, despite retention efforts: nurses were compensated for their completion of these activities, provided with reminders and encouragement to continue by research staff, and that activities could be completed via any computer or smartphone and location of their choosing so long as these devices had access to the internet. It may be that recruitment and retention was negatively impacted by stress and circumstances of the COVID-19 pandemic, which was ongoing during the recruitment and study period.

### Limitations and Considerations

As a formative evaluation of the eLearning training, this pilot study was designed to examine feasibility and acceptability and provide considerations for iterations on the training. Although we explored learning outcomes associated with the training as part of these considerations, the study was not designed to provide causal evidence of the effect of the training. In addition, some participants were lost at follow-up, further reducing the sample size.

Although nurses from across inpatient units and floors at the hospital could be participants in the study, the sample only comprised nurses from units who predominantly serve traumatically injured (including burns) patients. This could reflect variability in distribution of the study information and flyer by unit, interest of nurses in receiving the training, or availability of nurses to participate in a volunteer study that was designed to require 6 to 7 hours of time over 6 months. The study findings are limited in generalizability to the hospital and service setting the nurses work in, which has a preexisting emphasis on suicide prevention: the state requires suicide prevention continuing education for state licensure and in a hospital that engages in a universal suicide risk screening practice. Furthermore, nurses working in trauma care serve patients on their units who have been admitted due to an injury incurred during a suicide attempt. Our sample also predominantly identified as White and female, so our results may not generalize to nurses who identify as men, nonbinary, or transgender or nurses who identify as multiracial, Hispanic or Latino, or Black, Indigenous, or people of color. Percentages of female- and White-identifying nurses we observed are similar to those observed nationally in the 2020 National Nursing Workforce Survey, in which men account for 9% of the registered-nurse workforce and 81% identify as White [83].

There are limitations to some of the measures used in this study, as most were developed specifically for this study and do not have known psychometric properties; however, the knowledge and confidence measures developed demonstrated good internal consistency reliability. The preexisting measures used were the SUS and the SPIRS. The SUS is a well-validated scale and demonstrated good internal consistency reliability in our study. The SPIRS is an unpublished rating system for the Suicide Safety Planning Intervention. The data were coded by a single rater, the first author (DD), who was trained to reliably code with the developers of the scale. Although examining learning outcomes using observational coding by a trained rater as well of self-report is a strength of this study, for a future evaluation, having a second rater would increase the rigor of the design [84]. We similarly sought to examine training engagement through self-report and objective data from the Lyssn.io platform. We were able to obtain data on how many times nurses used Client Bot Emily over the course of the study but were unable to capture these data for Lyssn Advisor. The system does routinely capture when a video or audio recording is uploaded by a user, but for practical purposes, the study team did that on behalf of participants, so we were unable to confirm nurses had accessed the platform with use data. More granular objective data on use from the platform may be valuable for a larger evaluation to explore the relationship between the amount and patterns of technology use and its association with learning outcomes.

A final consideration is that training is only one step in the complex process of implementation of a new practice. Previous research with acute and intensive care nurses suggests there is a need and opportunity to engage patients at risk of suicide who are hospitalized for medical reasons in suicide prevention interventions; however, this setting also presents barriers to doing so [19]. Primary barriers include the busy hospital setting in which nurses face competing priorities and limited uninterrupted time to talk with patients about the sensitive topic of suicide. Patients’ abilities to engage also vary over the hospital stay; for instance, patients are often in pain, taking opioid medications, recovering from surgery, or otherwise experiencing alterations in their cognition and mental status. It may be that in actual practice nurses may need to deliver suicide safety planning with modifications, such as supporting patients to effectively use computer technology that guides them through the safety planning process as opposed to relaying solely on the nurse to complete the full intervention [85]. Either way, it remains valuable for nurses to receive training that increases their comfort, confidence, and skills in providing suicide-related care to patients.

### Conclusions

Integrating suicide prevention in medical settings and training health care workers in suicide prevention skills is a key part of the US National Strategy [11] to prevent suicide. Efficient, accessible, and effective continuing education trainings that can be brought to scale and meet this goal are needed. Novel computer and web-based technologies now make it possible to create eLearning continuing educational opportunities that use evidence-based learning methods. This study contributes uniquely to the training of nurses in the inpatient acute and intensive medical care context and the feasibility and acceptability of using 2 novel technologies relying on artificial intelligence to provide nurses with opportunities to practice suicide prevention skills and receive feedback on these skills. The use of these technologies is promising for future training in skills for suicide safety planning. Technologic modifications that may enhance the training include increasing the virtual patient conversational abilities and adding automated coding capability for specific suicide safety planning skills. A subsequent fully-powered trial of the eLearning training incorporating these modifications could then evaluate the impact on learning outcomes both in the training context and in routine care with patients.

## Appendix

Multimedia Appendix 1 Supplementary tables and figures.

## Abbreviations

REDCap: Research Electronic Data Capture
SPIRS: Safety Plan Intervention Rating Scale
SUS: System Usability Scale
WISE: Workplace Integrated Support and Education
