# Supplementary material for: Harnessing Innovative Technologies to Train Nurses in Suicide Safety Planning With Hospital Patients: Formative Acceptability Evaluation of an eLearning Continuing Education Training
Source: JMIR Form Res. 2024 Sep 6;8:e56402. doi: 10.2196/56402 (PMC11415721; doi:10.2196/56402)
Supplement: Multimedia Appendix 1 [file formative_v8i1e56402_app1.pdf]

Table S1. Description of the standardized patient role-play scenarios and responses for the safety planning steps

	Post-didactic role-play: Emelia	Role-play coded by Lyssn Advisor: Natalie	Post-training role-play: Maureen	6 month follow-up role-play: Brianna
**Demographics**	**Age:** 40 Female<br>**Gender:** Female<br>**Background:** Lives alone. Works at a dental office. Has a supportive girlfriend (partner) and mother. Mother experiences depression. Patient experiences periods of depression and attempted suicide one year previous. Patient worries about being a burden to her girlfriend and mother. Feels she has been doing "pretty well" with her mental health prior to the accident and is seeing a psychotherapist weekly since the suicide attempt.	**Age:** 20s<br>**Gender:** Female<br>**Background:** Lives with a roomate. Works part-time at an art supply store, but looking for full-time work. Had a recent breakup with boyfriend (partner) and emotional pain of this led to an interrupted suicide attempt three months prior. The break-up led to a move to a new apartment and increased financial concerns. Has a history of feeling abandoned by family. Has a new psychotherapist.	**Age**: 60s<br>**Gender:** Female<br>**Background:** Widowed for 2 years. Lives in a rural area and owns horses. Experienced a natural disaster, a landslide in her neighborhood, recently, although her home was not affected. Is facing unexpected early retirement due to a layoff at work and has significant financial concerns. Has no history of prior mental health treatment.	**Age:** Late teens<br>**Gender:** Female<br>**Background:** College freshman. Lives in a dorm at college, but hopes to join a sorority and may live there. Has a history of perfectionism and anxiety since middle school. Experienced some suicidality as a younger teenager. Never saw a professional for mental health reasons. Describes her parents as supportive and as her "life-coaches"; however, also feels pressure to meet their expectations.
**Reason in the hospital**	Car accident (not a suicide attempt)	Burns sustained in accidental kitchen fire	Broken hip sustained from an accidental fall	Suicide attempt by pain medication overdose
**C-SSRS screener**	**Risk Level:** Moderate<br>**Suicide Severity:** Current passive suicidality with no plan or intent; history of suicide attempt as a teenager and within the previous year in which patient ingested a bottle of prescribed antidepressant medication.	**Risk Level:** High<br>**Suicide Severity:** Current active suiciality with no intent but a vague plan. Experienced an interrupted suicide attempt within the previous 3 months using a box cutter her ex-boyfriend left behind that he used in his move out of their apartment.	**Risk Level:** Moderate<br>**Suicide Severity:** Current active suidicality. Has considered using a firearm she owns to kill herself but has not been taking steps to plan how she would do this and has no intent to kill herself. She has not made any prior suicide attempts.	**Risk Level:** High<br>**Suicide Severity:** Current active suicidality. In the hospital for a suicide attempt. Ingested opioid medications she found at her parents' house when visiting them.
**Warning signs**	**General context:**<br>Exhaustion and depression<br>**Specific thoughts and feelings:**<br>• Feeling exhausted with life<br>• Feeling like a burden and that she would be better off dead than burdening her family<br>• Thinking she is a loser and that her life is meaningless and worthless	**General context:**<br>Feelings of abandonment<br>**Specific thoughts and feelings:**<br>• Being alone in her apartment and feeling abandoned<br>• Thinking that there must be something wrong with her that makes people leave her	**General context:**<br>Grief, loneliness, and financial insecurities<br>**Specific thoughts and feelings:**<br>• Grief and overwhelming sense of loss, which is greatest in the evening when at home<br>• Feeling that life is empty and without joy<br>• Worrying about finances and thinking she will not be able to support herself	**General context:**<br>Perfectionism, anxiety, fear of disappointing parents<br>**Specific thoughts and feelings:**<br>• Feeling anxious about not meeting her parents' expectations due to poor grades<br>• Feeling ashamed and like a failure<br>• Thinking she is defective and imperfect, especially when comparing herself to others, which is heighened when using social media
**Internal coping strategies**	**Activities:**<br>• Listening to podcasts on uplifting topics<br>• Walking outside or indoors on treadmill	**Activities:**<br>• Watching old television sitcoms<br>• Dancing, when able to physically<br>• Artistic drawing or sketching	**Activities:**<br>• Reading uplifting books<br>• Listening to gospel music<br>• Spending time with the horses in her stable	**Activities:**<br>• Crossword puzzles<br>• Artistic outdoor photography<br>• Running for physical activity

Table S1. Description of the standardized patient role-play scenarios and responses for the safety planning steps

	Post-didactic role-play:<br>Emelia	Role-play coded by Lyssn Advisor: Natalie	Post-training role-play:<br>Maureen	6 month follow-up role-play:<br>Brianna
**People and social settings to provide distraction**	**People:**<br>• Mother<br>• Girlfriend<br>**Social settings:**<br>• Going to the public market to browse the shops	**People:**<br>• Roomate<br>• Father<br>**Social settings:**<br>•Taking sketch pad to the local park, coffee shop or art gallery	**People:**<br>• Neighbor<br>**Social settings:**<br>• Going to the grocery store<br>• Going to a movie<br>• Going to church	**People:**<br>• High school best friend<br>**Social settings:**<br>• Going to the library to study<br>• Going to a coffee shop with friends<br>• Running at the school track
**Friends or family to ask for help**	**People:**<br>• Mother<br>• Girlfriend	**People:**<br>• Roommate<br>• Father<br>• College professor	**People:**<br>• Cousin<br>• Church elder<br>• Coworker	**People:**<br>• High school best friend<br>• High school guidance counselor<br>• Parents
**Professionals or agencies to contact during a crisis**	• Psychotherapist<br><br>**Suicide prevention lifeline concern:**<br>Uncomfortable talking to a stranger about her mental health or suicidal thoughts but would be open to a text message line<br><br>**Mental health treatment concern:**<br>None	• Psychotherapist<br>• Local Urgent Care or Emergency Room<br><br>**Suicide prevention lifeline concern:**<br>None<br><br>**Mental health treatment concern:**<br>Struggles to find a good pychotherapist match and does not feel secure in current therapy relationship	• Call 911<br>• Local Urgent Care<br><br>**Suicide prevention lifeline concern:**<br>None<br><br>**Mental health treatment concern:**<br>• Does not want to see a psychotherapist as she feels like she should be able to handle problems on her own<br>• Rural area with limited care available locally<br>• Primary care doctor recently deceased in landslide	• Primary care doctor<br>• Campus medical clinic<br>• Local Urgent Care or Emergency Room<br><br>**Suicide prevention lifeline concern:**<br>None<br><br>**Mental health treatment concern:**<br>None

Table S1. Description of the standardized patient role-play scenarios and responses for the safety planning steps

	Post-didactic role-play: Emelia	Role-play coded by Lyssn Advisor: Natalie	Post-training role-play: Maureen	6 month follow-up role-play: Brianna
**Making the environment safe**	**Hesitancy:**<br>None<br>**Agreeable to do for means safety:**<br>• To keep a minimal amount of prescription and over the counter medications at home<br>• To not stockpile medications<br>• To keep medications in a locked cabinet<br>• To have her girlfriend assist with storage and dispensing of medications	**Hesitancy:**<br>• Does not want to getting rid of the box cutter because it belongs to her ex-boyfriend<br>**Agreeable to do for means safety:**<br>• To remove and dispose of the blade from the box cutter<br>• To not purchase any new blades or other sharp objects that she could cut herself with<br>• To keep needed sharp objects in locked drawers and access only when roommate is nearby<br>• To reduce the need to use sharp kitchen knives when experiencing suicidality (eg, purchase pre-cut produce, meats, etc.)	**Hesitancy:**<br>• Uses the firearms for home protection, so is hesitant to store in a way that limits access to loaded firearms<br>**Agreeable to do for means safety:**<br>• To get a security alarm service to reduce reliance on firearms for home protection, and then store firearms in a locked safe or unloaded and separate from ammunition<br>• To keep a limited amount of over the counter and prescription medications at home	**Hesitancy:**<br>None<br>**Agreeable to do for means safety:**<br>• To ask her parents to keep their prescription medications locked or hidden when she visits their home<br>• To keep travel-sized amounts of over the counter medication available
**Reason for living**	Does not want to cause her mother or girlfriend pain by killing herself	Does not want to cause her father pain by killing herself	• Suicide is against her religious beliefs<br>• Does not want to disappoint her deceased husband<br>• Does not want to leave her horses	• Does not want to disappoint her parents<br>• Wants to fulfill her dream of becoming a lawyer and help others

Note. C-SSRS = Columbia Suicide Severity Rating Scale. Responses for the safety planning steps align with the Suicide Safety Planning Intervention with the addition of reasons for living.

Table S2. Information and instructions provided to nurses in written format prior to completing each role-play

	Post-didactic role-play: Emelia			Role-play coded by Lyssn Advisor: Natalie			Post-training role-play: Maureen			6 month follow-up role-play: Brianna		
**Scenario description**	Emelia is a hospitalized patient who was admitted to a surgical floor after sustaining injuries due to a car accident. She was screened for suicide risk using the Columbia triage screener. On the screener she noted current passive suicidality (with no plan or intent) and has a history of suicide attempts. Her most recent attempt was a little over a year ago. She attempted to overdose by taking a full bottle of medications she was prescribed for depression by her primary care provider. She started seeing a psychotherapist after this and is engaged in weekly therapy.			Natalie is a hospitalized patient who was admitted to the burn unit after sustaining injuries from a kitchen fire. She was screened for suicide risk using the Columbia triage screener. She was screened on the Columbia after she was discharged from ICU. On the screener she noted current suicidality (with no intent) and vague plan, and has a history of suicide attempts. 3 months ago she seriously contemplated suicide, and was sitting in her bathtub with a box cutter blade thinking about what it would be like to cut her wrists, but was interrupted by her roommate. Natalie has been struggling to find a psychotherapist she feels comfortable with, and recently started weekly therapy sessions with someone new.			Maureen is a hospitalized patient who is recovering from a recent hip replacement surgery following a fall that fractured her hip (tripped on uneven sidewalk). She was screened for suicide risk using the Columbia triage screener. On the screener she noted current suicidality. No prior attempts, but has been contemplating ending her life, has access to a gun, and has wondered if she could use it to end her life. Maureen has not been seeing a psychotherapist and never had mental health treatment of any kind.			Brianna is a hospitalized patient admitted to an acute care unit following a suicide attempt. She was screened for suicide risk using the Columbia triage screener. On the screener she notes current suicidality and that she recently attempted to end her life when she was home from college on a break by overdosing on her mother's prescription pain medication. Brianna has no prior history of suicide attempt, however, has experienced perfectionism and anxiety since middle school. She has never sought mental health counseling as she relies on her parents as her "life-coaches."		
**C-SSRS Screener**		Yes	No		Yes	No		Yes	No		Yes	No
		Past Month			Past Month			Past Month			Past Month	
	1) Have you wished you were dead or wished you could go to sleep and not wake up?	X		1) Have you wished you were dead or wished you could go to sleep and not wake	X		1) Have you wished you were dead or wished you could go to sleep and not wake up?	X		1) Have you wished you were dead or wished you could go to sleep and not wake up?	X	
	2) Have you had any actual thoughts of killing		X	2) Have you had any actual thoughts of	X		2) Have you had any actual thoughts of killing	X		2) Have you had any actual thoughts of killing	X	
	3) Have you been thinking about how you might do this?	N/A	N/A	3) Have you been thinking about how you might do this?	X		3) Have you been thinking about how you might do this?	X		3) Have you been thinking about how you might do this?	X	
	4) Have you had these thoughts and had some intention of acting on them?	N/A	N/A	4) Have you had these thoughts and had some intention of acting on them?		X	4) Have you had these thoughts and had some intention of acting on them?		X	4) Have you had these thoughts and had some intention of acting on them?	X	
	5) Have you started to work out or worked out the details of how to kill yourself? Do you	N/A	N/A	5) Have you started to work out or worked out the details of how to kill yourself? Do		X	5) Have you started to work out or worked out the details of how to kill yourself? Do you		X	5) Have you started to work out or worked out the details of how to kill yourself? Do you	X	
		Lifetime			Lifetime			Lifetime			Lifetime	
	6) Have you ever done anything, started to do anything, or prepared to do anything to end your life?	X		6) Have you ever done anything, started to do anything, or prepared to do anything to end your life?	X		6) Have you ever done anything, started to do anything, or prepared to do anything to end your life?		X	6) Have you ever done anything, started to do anything, or prepared to do anything to end your life?	X	
		Past 3 Months			Past 3 Months			Past 3 Months			Past 3 Months	
	If yes, was this within the past 3 months?		X	If yes, was this within the past 3 months?	X		If yes, was this within the past 3 months?		X	If yes, was this within the past 3 months?	X	
**C-SSRS Score**	Moderate			High			Moderate			High		
**Suggested script to start the role-play**	Hi Emelia. If you remember, we talked earlier that at times in the past things became really challenging for you and this led you to attempt suicide [discussed during Columbia screening, for instance]. We are offering a service to our patients that have had suicidal thoughts and I am hoping we could spend a few minutes together now on this.			Hi Natalie. If you remember, we talked earlier that at times in the past things became really challenging for you and this led you to attempt suicide [discussed during Columbia screening, for instance]. We are offering a service to our patients that have had suicidal thoughts and I am hoping we could spend a few minutes together now on this.			Hi Maureen. If you remember, we talked earlier that at times in the past things became really challenging for you, and you mentioned having suicidal thoughts [discussed during Columbia screening, for instance]. We are offering a service to our patients that have had suicidal thoughts and I am hoping we could spend a few minutes together now on this.			Hi Brianna. We offer a service to our patients that have had suicidal thoughts or came into the hospital after a suicidal attempt and I am hoping I could spend a few minutes with you now to talk about this service.		

Note. C-SSRS = Columbia Suicide Severity Screener. For all role-plays, nurses were also provided the instruction, "If you aren't sure what to do, you can do whatever you would normally do to be helpful to a patient who expresses suicidal thoughts."

Table S3. Knowledge and confidence questions for suicide safety planning

Obtain a narrative of a crisis and provide a rationale for suicide safety planning	
1	Obtain a narrative description from the patient about an experience of a suicidal crisis
2	Use the narrative description to help the patient identify warning signs that led to the suicidal crisis
3	Use the narrative description to illustrate how suicidal thoughts and urges change over time
4	Provide a rationale for how coping strategies can help the patient get safely through a suicidal crisis
5	Provide a rationale for how having sources of social or professional support in place can help the patient with managing the crisis
Engage in safety planning	
6	Work collaboratively with the patient (to develop their safety plan)
7	Provide a rationale for using warning signs as part of the patient's safety plan
8	Help the patient identify specific and individualized warning signs of a suicidal crisis
9	Provide a rationale for how distraction strategies can help the patient get safely through a suicidal crisis
10	Help the patient identify distraction strategies that they can practically do on their own.
11	Address barriers to using these distraction strategies
12	Provide a rationale for how social activities or interacting with other people to distract can help the patient get safely through a suicidal crisis
13	Help the patient identify people or healthy social settings that can provide a distraction
14	Getting actual phone numbers for social support contacts to write down on the safety plan
15	Address barriers to using these socially-oriented distraction strategies
16	Provide a rationale for why reaching out to trusted friends or family can help the patient get safely through a suicidal crisis
17	Help the patient identify trusted friends or family who the patient can realistically contact for help through a crisis
18	Getting actual phone numbers for social support contacts to write down on the safety plan
19	Address barriers to contacting the friends or family identified as being sources of support during a suicidal crisis
20	Provide a rationale for why reaching out to professionals, agencies, or emergency services can help the patient get safely through a suicidal crisis
21	Help the patient identify professionals, agencies, or emergency services that the patient could realistically contact for help through a crisis
22	Getting actual phone numbers for professionals/agency contacts to write down on the safety plan
23	Address barriers to contacting these professionals/agencies if going through a suicidal crisis
24	Provide a rationale as to why reducing access to lethal means can help the patient get safely through a suicidal crisis
25	Help the patient identify ways to reduce access to lethal means
26	Address barriers to carrying out the action plan for reducing access to lethal means
Describe how to use the safety plan	
27	Provide instruction on when to use the safety plan
28	Provide instruction on how to use the safety plan
29	Help the patient decide where or how to store the suicide safety plan

30	Help the patient decide who they can share the plan with
31	Address barriers to using the safety plan
32	Have a copy of the patient's suicide safety plan put into the EHR

Note. Nurses were asked to rate how knowledgeable they felt about each of the 32 aspects and how confident they were doing each in a role-play with a standardized patient on a Likert-type scale from with 0 = not at all, 1 = a little, 2 = somewhat, 3 = a good bit, and 4 = very.

Figure S1

Scatterplots and bivariate correlations for length of time nurses took to complete the role-play and the quality of the suicide safety planning intervention as scored by the Safety Plan Intervention Scale (SPIRS). Scatterplots and bivariate correlations are presented for each the role-plays: post-didactic, post-training, and 6-month follow-up role-plays. Scatterplots and bivariate correlations are presented for each of the SPIRS subscales: General (eg, asking the patient for a narrative of a recent suicidal crisis and providing a rationale for completing a safety plan) and Specific SPI skills (ie, 6 aspects corresponding to the steps outlined on the written safety plan).

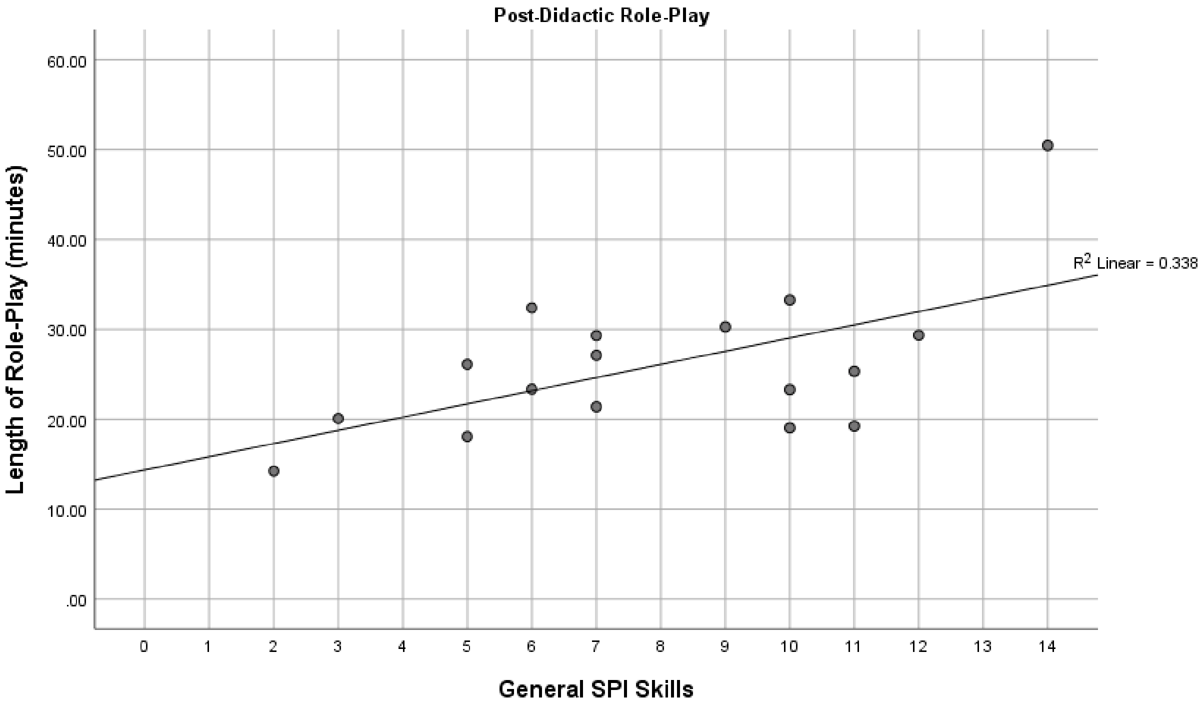

Bivariate correlation = 0.58, P = 0.01, *n* = 17

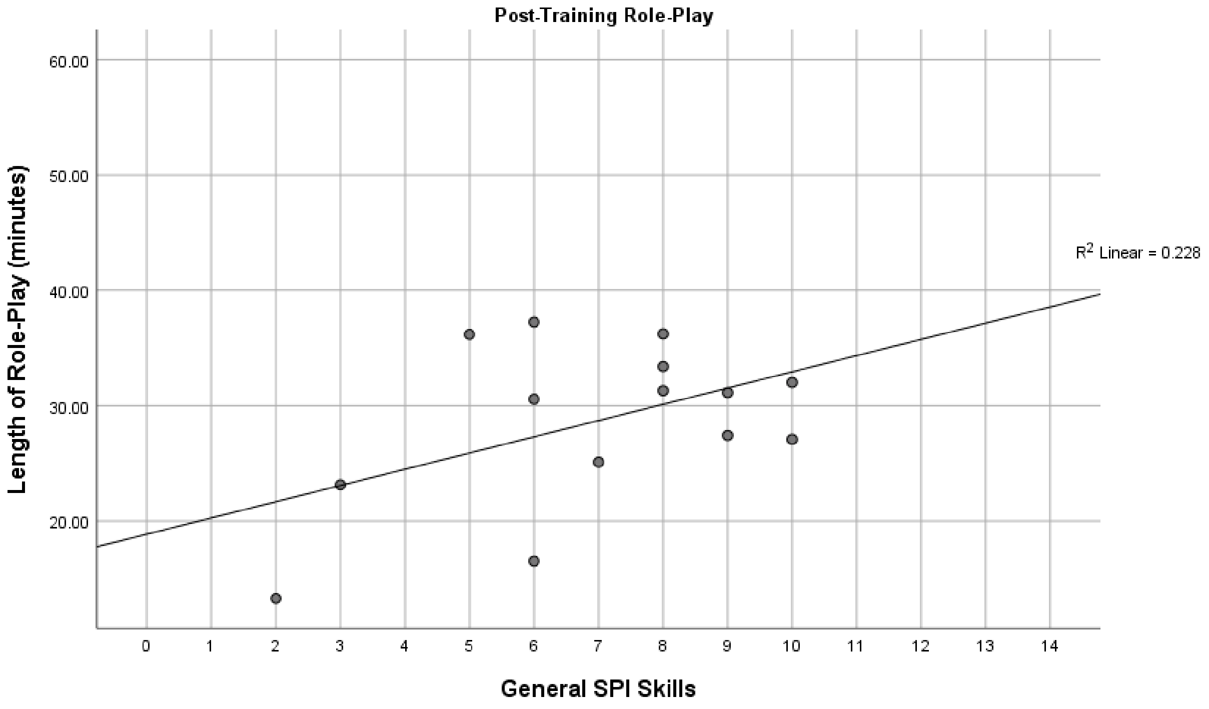

Bivariate correlation = 0.48, P = 0.08, *n* = 14

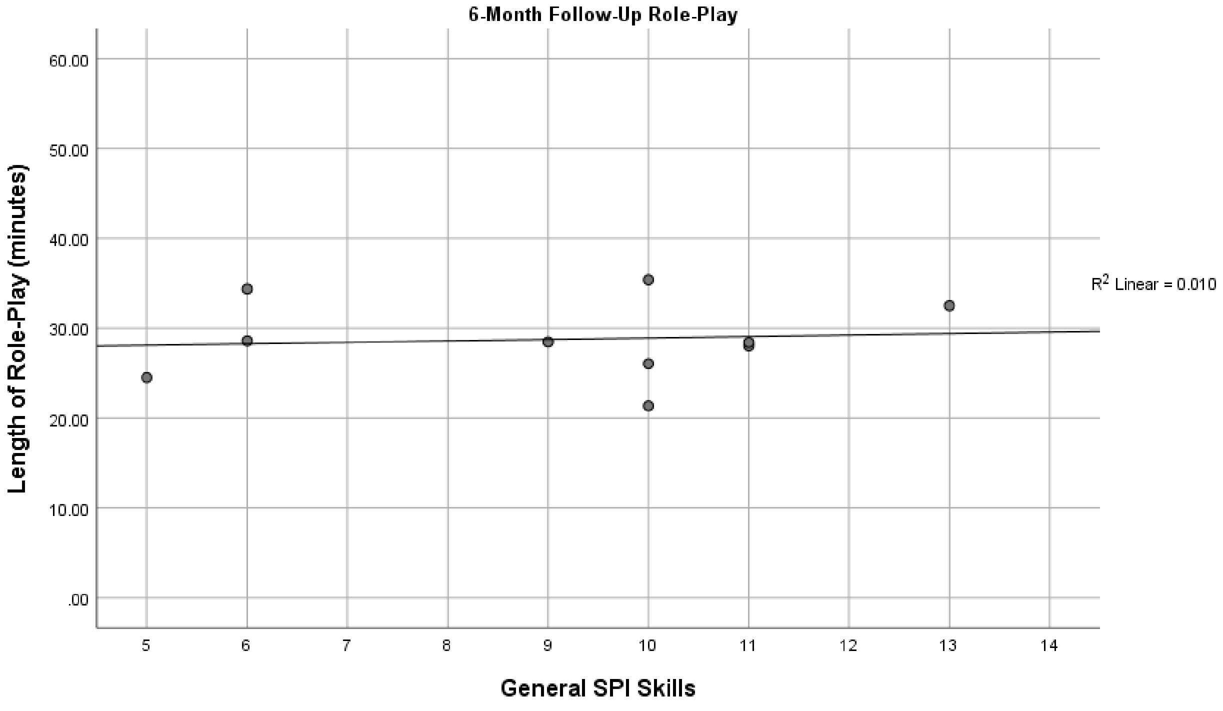

Bivariate correlation = 0.10, P = 0.79, *n* = 10

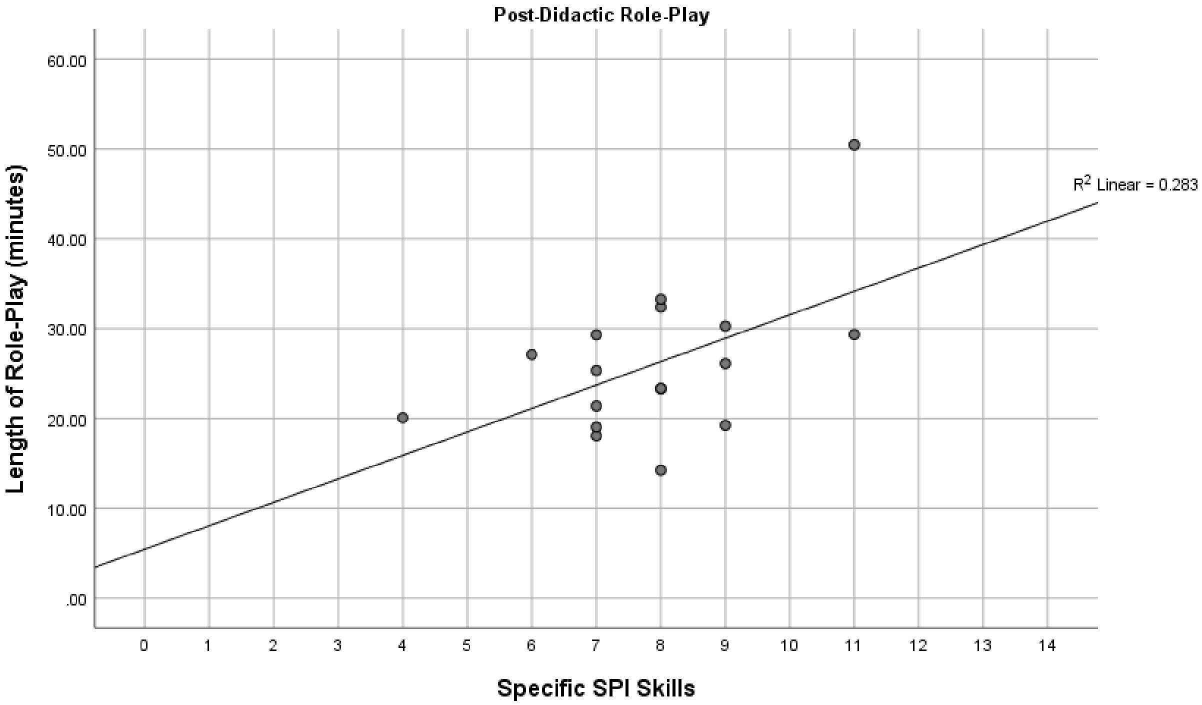

Bivariate correlation = 0.53, P = 0.03, *n* = 17

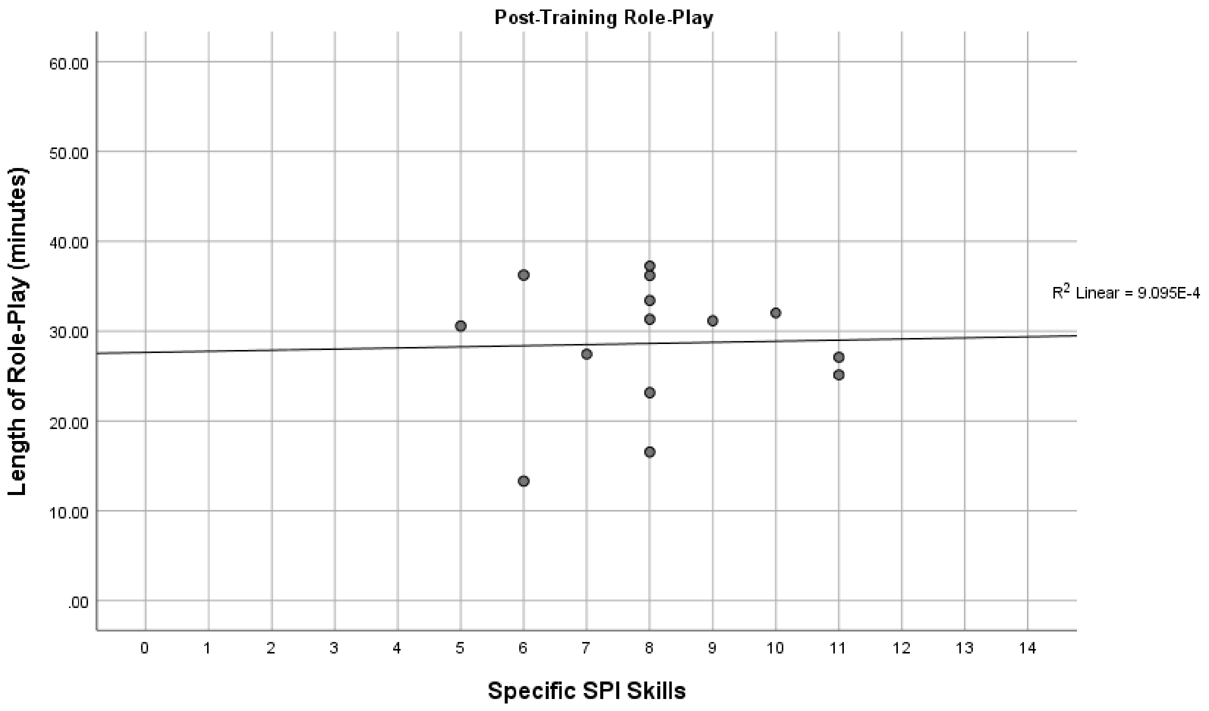

Bivariate correlation = 0.03, P = 0.92, *n* = 14

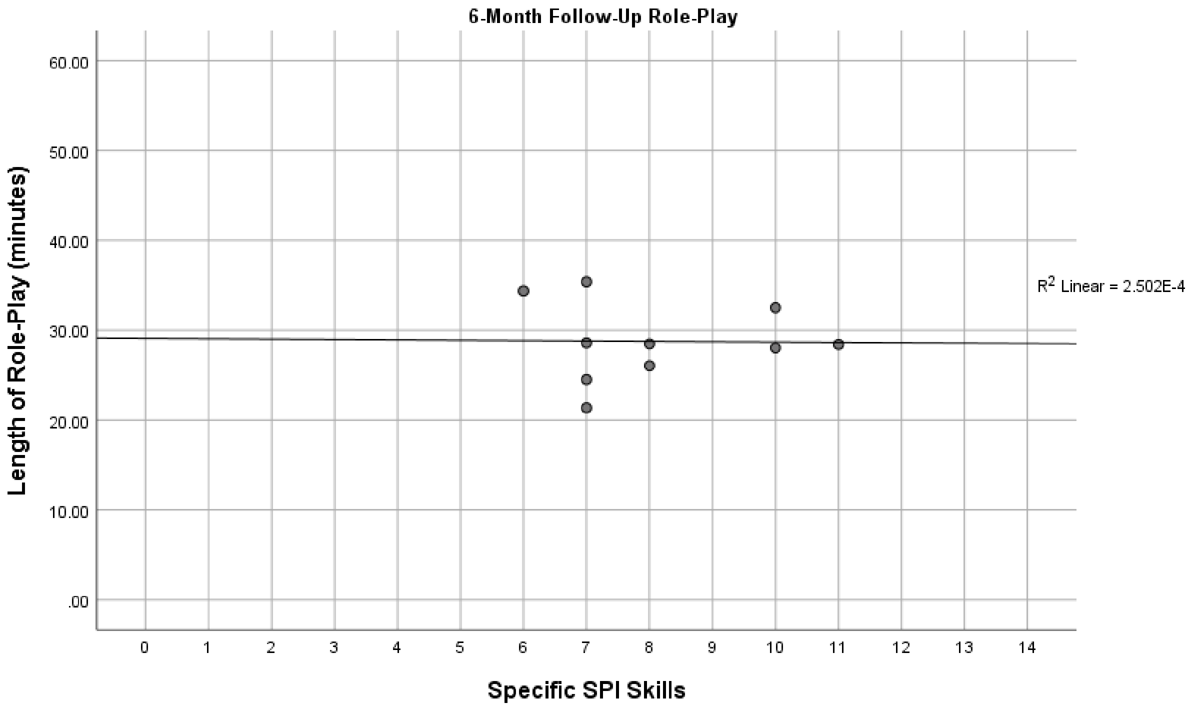

Bivariate correlation = -.16, P = 0.97, *n* = 10
